# Symmetry Breaking in Rationally Designed Copper Oxide Electrocatalyst Boosts the Oxygen Reduction Reaction

**DOI:** 10.1002/advs.202411928

**Published:** 2024-12-16

**Authors:** Haoyu Peng, Weiyi Wang, Jiyuan Gao, Fan Jiang, Bowei Li, Yicheng Wang, Yiqian Wu, Yue Wang, Jiuqiang Li, Jing Peng, Wei Hu, Zhenhai Wen, Dingsheng Wang, Erhuan Zhang, Maolin Zhai

**Affiliations:** ^1^ Beijing National Laboratory for Molecular Sciences Radiochemistry and Radiation Chemistry Key Laboratory of Fundamental Science The Key Laboratory of Polymer Chemistry and Physics of the Ministry of Education College of Chemistry and Molecular Engineering Peking University Beijing 100871 P. R. China; ^2^ Future Battery Research Center Institute of Future Technology Shanghai Jiao Tong University Shanghai 200240 P. R. China; ^3^ Hefei National Research Center for Physical Sciences at the Microscale Department of Chemical Physics University of Science and Technology of China Hefei 230026 P. R. China; ^4^ CAS Key Laboratory of Design and Assembly of Functional Nanostructures Fujian Provincial Key Laboratory of Nanomaterials Fujian Institute of Research on the Structure of Matter Chinese Academy of Sciences Fuzhou 350002 P. R. China; ^5^ Future Photovoltaic Research Center Global Institute of Future Technology Shanghai Jiao Tong University Shanghai 200240 P. R. China; ^6^ Department of Chemistry Tsinghua University Beijing 100084 P. R. China

**Keywords:** copper oxide catalyst, electrocatalysis, irradiation synthesis, local broken‐symmetry, oxygen reduction reaction

## Abstract

Oxygen reduction reaction (ORR) kinetics is critically dependent on the precise modulation of the interactions between the key oxygen intermediates and catalytic active sites. Herein, a novel electrocatalyst is reported, featuring nitrogen‐doped carbon‐supported ultra‐small copper oxide nanoparticles with the broken‐symmetry *C*_4v_ coordination filed sites, achieved by a mild γ‐ray radiation‐induced method. The as‐synthesized catalyst exhibits an excellent ORR activity with a half‐wave potential of 0.873 V and shows no obvious decay over 50 h durability in alkaline solution. This superior catalytic activity is further corroborated by the high‐performance in both primary and rechargeable Zn–air batteries with an ultrahigh‐peak‐power density (255.4 mW cm^−2^) and robust cycling stability. The experimental characterizations and density functional theory calculations show that the surface Cu atoms are configured in a compressed octahedron coordination. This geometric arrangement interacts with the key intermediate OH^*^, facilitating localized charge transfer and thereby weakening the Cu─O bond, which promotes the efficient transformation of OH^*^ to OH^−^ and the subsequent desorption, and markedly accelerates kinetics of the rate‐determining step in the reaction. This study provides new insights for developing the utilization of γ‐ray radiation chemistry to construct high‐performance metal oxide‐based catalysts with broken symmetry toward ORR.

## Graphical abstract

A copper oxide‐based catalyst featuring nitrogen‐doped carbon‐supported ultra‐small sizes and broken‐symmetry *C*_4v_ sites, achieved by a γ‐ray radiation‐induced method under ambient conditions. The as‐synthesized catalyst exhibits excellent ORR activity in alkaline solution which is further corroborated by the high‐performance in both primary and rechargeable Zn–air batteries. Experimental characterizations and DFT calculations show that the surface Cu atoms are configured in a compressed tetrahedral coordination. This geometric arrangement interacts with the key intermediate OH^*^, facilitating localized charge transfer and thereby weakening the Cu─O bond, which promotes the efficient transformation of OH^*^ to OH^−^ and the subsequent desorption, and markedly accelerates kinetics of the rate‐determining step in the reaction.

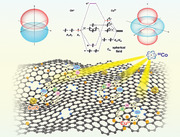

## 1 Introduction

Oxygen reduction reaction (ORR) is essential for the operation of electrochemical energy storage and conversion devices, such as fuel cells and metal‐air batteries.^[^ ^1^, ^2^, ^3^, ^4^, ^5^ ^]^ However, achieving ORR electrocatalysts with both high catalytic activity and cost‐effectiveness remains challenging due to the inherently sluggish kinetics of O_2_ reduction.^[^ ^6^, ^7^, ^8^ ^]^ Currently, platinum‐group‐metal (PGM)‐based materials are considered to be the most promising catalysts, whereas their limited reserves and costs significantly constrain their scalability and widespread application for the practical commercialization.^[^ ^8^, ^9^, ^10^, ^11^ ^]^ Hence, it is highly necessary to develop the abundant and low‐cost materials toward ORR with excellent activity and stability. To realize these objectives, we should create a comprehensive strategy for the rational design of catalytic structures and to employ advanced fabrication techniques in the development of catalytic materials.

In the design of ORR materials, it is crucial to both improve the intrinsic activity of catalytic sites and optimize the geometric configurations to maximize the site exposure and facilitate efficient mass transfer.^[^ ^12^, ^13^, ^14^ ^]^ From a structural perspective, the interaction between the transition metal and the carbon element can facilitate the high intrinsic catalytic activity. Recent studies have demonstrated optimize the catalytic activity of porous carbon‐based materials. Further modulating the interfacial interaction and coordination/electronic environment via a “breaking the symmetry” strategy can enhance the electron transfer behavior during the electrocatalytic reactions.^[^ ^15^, ^16^, ^17^, ^18^, ^19^, ^20^, ^21^, ^22^ ^]^ To control the metal nanoparticles (NPs), metal oxide, or atomically dispersed metal from the reduction of metal ions in the metal–organic frameworks (MOFs), can effectively disperse and stabilize metal‐based sites on carbon substrates.^[^ ^3^, ^15^, ^16^, ^23^, ^24^ ^]^ Substituting part of the coordinated M‐N_4_ moiety with other coordinated atoms, such as O, S, P, and B, will break the coordination symmetry, increase the site polarity, and generates an unsaturated rupture with localized excess charges, which may have a positive effect on the electrocatalytic activity.^[^ ^14^, ^23^, ^24^, ^25^, ^26^, ^27^ ^]^ Except for the broken single‐atom site of various axial ligands, intrinsic local symmetry breaking in transition metal oxide for electrocatalytic reactions has not been reported. The coordination environment of Cu^2+^ on the surface of CuO NPs approximates the *C*_4v_ crystal field of a compressed octahedron due to symmetry‐reduced Jahn‐Teller distortion along one of the four‐fold axes.^[^ ^28^, ^29^, ^30^ ^]^ The higher energy $d_{z^{2}}$ generated by *d* orbital splitting is partially occupied, which will form σ^*^ orbital with the key intermediate OH^*^ of the oxygen reduction reaction, reduce the bonding strength of OH^*^ on Cu(II) and improve the ORR kinetics.^[^ ^31^, ^32^, ^33^ ^]^ On the basis of understanding the catalytic mechanism, it is feasible to control the electronic structure of the active center and optimize the reaction path by precisely constructing CuO NPs catalysts supported on doped porous carbon. However, due to the large diffusion coefficient of copper ions, the commonly prepared copper oxide materials are prone to self‐aggregation and irreversible integration, inhibiting the improvements in their electrocatalytic performance.^[^ ^34^, ^35^ ^]^ To circumvent these issues, gamma(γ)‐rays could promote the conversion of high oxidation state metal ions in the solution to obtain nano‐scale metallic elemental or metal oxides in the solution and benefit to the preparation of well‐dispersed supported NPs catalysts thanks to their high energy and penetration.^[^ ^36^, ^37^ ^]^ For example, ^60^Co γ‐ray source can uniformly trigger the generation of solvated electrons (e_sol_^−^), hydrogen radicals (·H), hydroxyl radicals (·OH), and other reactive species in aqueous solutions.^[^ ^38^, ^39^, ^40^, ^41^, ^42^ ^]^ These active species can provide sufficient energy to promote the chemical synthesized reactions, allowing for the precise transformation of precursors through accurate control of the dose rate and absorbed dose. Compared with liquid‐phase synthesis and high‐temperature pyrolysis, the radiation method can enable the synthesis of materials under milder conditions without the use of strong oxidizing, reducing, or toxic reagents.^[^ ^36^, ^38^, ^39^, ^40^, ^41^ ^]^ Therefore, it is desired to establish a γ‐ray‐driven strategy to achieve very well‐defined CuO‐based nanomaterials with a high ORR activity.

In this work, we propose a novel and efficient strategy for catalyst fabrication. Cu^2+^ ions are successfully in situ converted into CuO NPs and then uniformly dispersed on the MOFs‐derived nitrogen‐doped porous carbon (CuO/NC) by the γ‐ray irradiation method. The CuO/NC‐based electrode achieved a superior ORR activity with an onset potential of 0.95 V and a half‐wave potential of 0.873 V in 0.1 m KOH electrolyte. Comprehensive experimental characterizations and density functional theory (DFT) calculations reveal that the CuO/NC possesses a compressed tetrahedral coordination configuration. This structure enhances the interaction between surficial Cu atoms and key intermediate OH^*^, resulting in localized charge transfer to OH^*^, the rapid conversion of OH^*^ to OH^−^ and subsequent efficient desorption. Further assembled primary and rechargeable zinc–air batteries demonstrated the catalyst's excellent performance in accelerating ORR kinetics. This work illustrates the feasibility of constructing supported metal oxide catalysts with symmetry broken by γ‐ray radiation chemistry, and provides valuable insights into the relationship between electronic structure and catalytic activity.

## 2 Results and Discussion

**Figure 1:**
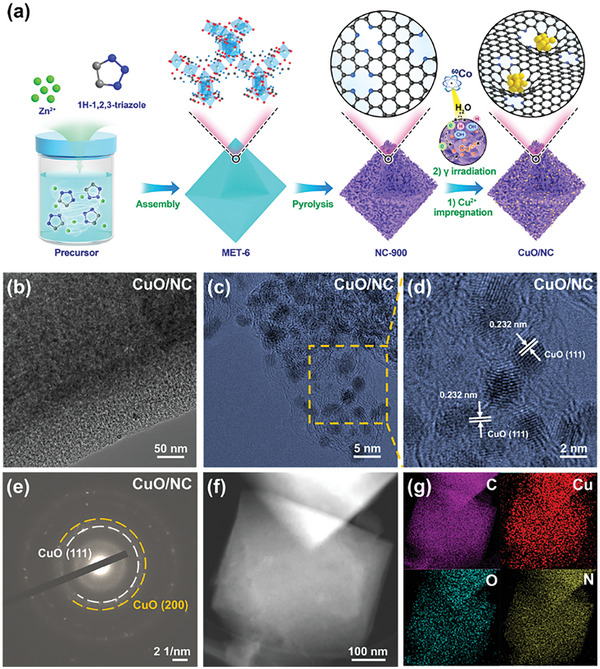
a) Schematic for the preparation of the resultant CuO/NC. b) TEM, c,d) HRTEM, e) SAED, f) STEM image, and g) the corresponding EDX mappings of the resultant CuO/NC.

The entire synthetic pathway of CuO/NC is illustrated in **Figure** **1**a (see more details in Supporting Information). Briefly, Zn‐based MOF (MET‐6) is synthesized through a one‐pot room‐temperature hydrothermal process. Cu^2+^ ions were subsequently impregnated and adsorbed onto a nitrogen‐doped carbon (NC‐900) framework, derived from the pyrolysis of the MET‐6 precursor. The species, generated through by γ‐ray initiation, actively participate in and provide energy required for the chemical conversion of Cu^2+^. During this process, Cu^2+^ are in situ converted into CuO NPs and further formed CuO/NC. Due to the strong penetration of γ‐rays, the chemical reaction can occur uniformly in the solution, which is beneficial to the preparation of the well‐defined CuO NPs in an ultra‐small size. The control sample (Cu/NC) is also prepared by adopting the copper (II) acetate monohydrate rather than Copper (II) chloride dihydrate in a similar mothed to that of CuO/NC, as seen details in the Experimental Section of Supporting information. As seen from the scanning electron microscope (SEM) images (Figures S1–S4, Supporting Information), MET‐6 exhibits an octahedral morphology with aggregate sizes ranging from 1.0 to 2.0 µm. After the pyrolysis and irradiation treatment, the morphologies and sizes are well‐maintained. According to the powder X‐ray diffraction (PXRD) results (Figure S5, Supporting Information), we cannot observe CuO or metallic Cu phases in CuO/NC and Cu/NC. This could be due to the extreme small sizes induced by irradiation. Raman spectra (Figure S6, Supporting Information) show that the intensity ratio of band D to band G (*I*_D_/*I*_G_) for CuO/NC, Cu/NC, and NC‐900 were 1.02, indicating that the carbon framework obtained from the pyrolysis of MET‐6 possessed an excellent irradiation stability and featured graphitic property with abundant defects.

Transmission electron microscopy (TEM) images (Figures S7, S10, Supporting Information) exhibit abundant mesopores structures for CuO/NC, Cu/NC, and NC‐900, measured by the nitrogen sorption isotherm experiments at 77 K. The CuO/NC exhibited a Brunauer–Emmett–Teller (BET) surface area and pore volume: 383 m^2^ g^−1^ and 0.89 cm^3^ g^−1^, respectively (Figure S8 and Table S1, Supporting Information). These abundant mesopore structures could promote the exposure of active sites and charge transport during the catalytic process.^[^ ^43^, ^44^ ^]^ As shown in Figures 1b,c, CuO NPs are semi‐embedded in the carbon framework. Additionally, Cu/NC showed a similar structure to that of CuO/NC (Figure S10, Supporting Information). The interplanar spacing of CuO NPs was determined by high‐resolution TEM (HRTEM, Figure 1d), in which the lattice with a spacing of 0.232 nm corresponds to the (111) plane of CuO.^[^ ^45^, ^46^, ^47^ ^]^ In the related selected area electron diffraction (SAED) patterns (Figure 1e), two sets of diffraction spots can be associated with the (111) and (200) crystal planes of cubic phase CuO, respectively.^[^ ^46^, ^48^ ^]^ Scanning transmission electron microscopy (STEM) images and corresponding energy‐dispersive X‐ray (EDX) elemental mapping showed the homogenous distribution of Cu, C, N, and O over the entire CuO/NC (Figure 1f,g). The size of CuO NPs is calculated to be 2.65 ± 0.60 nm (Figure S9, Supporting Information). Such ultra‐small size is consistent with the large specific surface area. These characteristics could be beneficial to the sufficient exposure of the catalytic active sites. The preparation of Cu/NC reference is also successfully obtained (Figure S10, Supporting Information).^[^ ^49^, ^50^, ^51^ ^]^

**Figure 2:**
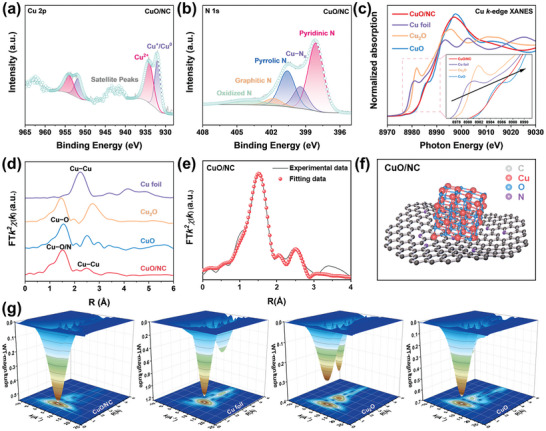
Chemical state and atomic local structural of CuO/NC: high‐resolution XPS a) Cu 2p and b) N 1s spectra. c) Cu K‐edge XANES spectra. d) *k* ^2^‐weighted Fourier transforms. e) χ(R) space spectra fitting curve of Cu K‐edge. f) Structural schematic diagram of CuO/NC. g) WT‐EXAFS plots of CuO/NC, Cu foil, Cu_2_O, and CuO.

The chemical states of Cu, N, and C in various samples were deciphered by the X‐ray photoelectron spectroscopy (XPS). The peaks corresponding to Cu species were observed in the XPS full spectra (Figure S11, Supporting Information). The CuO/NC sample is dominated by sp^2^ hybridization as shown in the high‐resolution C 1s spectrum (Figure S12, Supporting Information). This indicated the superior electron transfer capability of the nitrogen‐doped carbon frameworks derived from MET‐6.^[^ ^52^ ^]^ **Figure** **2**a shows the high‐resolution Cu 2p spectrum of CuO/NC, in which the peaks at binding energies (BEs) of 934.2 eV could be ascribed as Cu^2+^.^[^ ^53^ ^]^ As expected, the Cu LMM Auger spectrum confirmed the absence of metallic Cu^0^ (Figure S13, Supporting Information).^[^ ^53^ ^]^ In Figure 2b, the high‐resolution N 1s spectrum can be divided into pyridinic N (398.2 eV), Cu−N*_x_* (399.4 eV), pyrrolic N (400.6 eV), graphitic N (401.8 eV), and oxidized N (404.2 eV).^[^ ^54^, ^55^ ^]^ The relative content of pyridinic‐N in the resultant CuO/NC is higher than that of Cu/NC (47.66% vs 40.75%), which ​could contribute to the enhanced ORR kinetics. Pyridinic‐N species can serve as true ORR active sites to accelerate electron transfer and improve the limiting current density​. Moreover, the high electrostatic repulsion of pyrrole N (25.43% of total N species in Table S2, Supporting Information) directs its lone pair point toward the sp^2^ carbon plane, thus facilitating charge transport behaviors.^[^ ^56^ ^]^ The high‐resolution XPS spectra of Cu/NC sample revealed the formation of zero valence in Cu species with substantial nitrogen species (Figures S14, Supporting Information).^[^ ^52^ ^]^

To further investigate the CuO/NC configuration, we measure X‐ray absorption near edge structure (XANES) and extended X‐ray absorption fine spectrum (EXAFS) spectroscopy. Unlike Cu foil and Cu_2_O, Figure 2c shows the near‐edge absorption energy of CuO/NC closing to CuO. The oxide state of Cu in CuO/NC is around Cu (II) state. Additionally, the lower adsorption of CuO/NC energy indicated that the stacking of CuO on N‐doped carbon leads to an increase in the charge density of the Cu center. The local structural information around Cu is then examined by the Fourier‐transformed *k* ^2^‐weighted EXAFS (FT‐EXAFS). The FT‐EXAFS curves of CuO/NC sample (Figure 2d) showed two prominent peaks at ≈1.5 and 2.5 Å, which can be assigned to the first shell of Cu─O/N and Cu─Cu coordination scattering path, respectively.^[^ ^57^ ^]^ The wavelet transform (WT) Cu K‐edge EXAFS contour plot (Figure 2g) exhibits an intensity maximum at about (5.4 Å^−1^, 1.5 Å) for CuO/NC, assigned to the back‐scattering of Cu─O in the first shell.^[^ ^58^ ^]^ Quantitative least squares EXAFS curve fitting analysis (Figure 2e; Figures S15, S16, Supporting Information) shows that the O coordination number in CuO/NC is ≈ 4 with a bond length of 1.94 Å, whilst the coordination number of the Cu atom is ≈1 with the average Cu─Cu bond length of 2.91 Å (Table S3, Supporting Information), which indicate a coordination unsaturation of the surfacial Cu atoms. These results indicated the cubic Fm‐3m space group of CuO in CuO/NC (Figure 2f).

**Figure 3:**
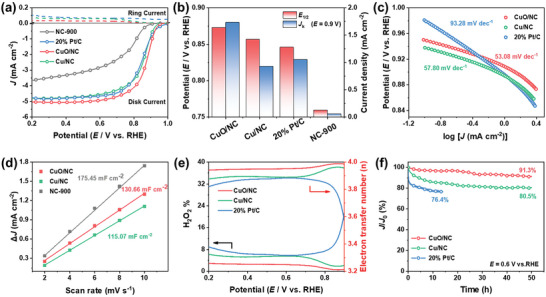
a) ORR polarization curves of CuO/NC, Cu/NC, Pt/C, and NC‐900 electrocatalysts. b) Corresponding *E*_1/2_ and *J*_k_ (*E* = 0.9 V) values. c) Corresponding Tafel plots. d) Electrochemical double‐layer capacitances (*C*_dl_). e) Electron transfer number (n, right) and H_2_O_2_ yield (left) of CuO/NC, Cu/NC, and Pt/C versus potential. f) Normalized chronoamperometry curves of CuO/NC, Cu/NC, and Pt/C at a constant potential of 0.6 V.

The electrocatalytic performance of the prepared CuO/NC catalyst toward ORR is evaluated using a typical four‐electrode measurement, recorded with a rotating ring‐disk electrode (RRDE) in Ar/O_2_‐saturated 0.1 m KOH aqueous solution, and compared with those of NC‐900, Cu/NC, and commercial Pt/C. ​All the potentials are calibrated with respect to the reversible hydrogen electrode (RHE) and corrected with iR‐compensation.​ The cyclic voltammetry (CV) curves show an obvious reduction peak at ≈0.8 V in the O_2_‐saturated solution while no corresponding response peak in the Ar‐saturated solution. This indicates that the CuO/NC exhibited an electrochemical activity toward ORR (Figure S17, Supporting Information). The intrinsic activity of ORR is preliminarily revealed by linear sweep voltammetry polarization curves. In **Figure** **3**a, CuO/NC shows the highest activity in terms of the most positive half‐wave potential (*E*_1/2_, 0.873 V), which is much more positive than the commercial Pt/C (0.846 V) and Cu/NC (0.857 V), respectively. The metal‐free NC‐900 exhibited inferior catalytic activity compared to those supported metal catalysts, suggesting the critical role of CuO/Cu species in ORR. As shown in Figure 3b, CuO/NC displays a remarkable kinetic current density (*J*_k_) of 1.734 mA cm^−2^ at 0.9 V, significantly higher than the commercial Pt/C (*J*_k_ = 1.057 mA cm^−2^) and Cu/NC (*J*_k_ = 0.932 mA cm^−2^). Based on electrochemical impedance spectra (EIS), the Nyquist plots of CuO/NC approximate to a quarter circle rather than an ideal semi‐circle shape. This is possibly caused by the faster charge transfer within the catalyst, making it difficult for mass transfer‐controlled processes to occur in the frequency range provided (e.g., from 0.1 Hz to 10 MHz). The fitted data show a reduced electron transfer resistance (*R*_ct_) of CuO/NC (40.59 Ω) compared to Cu/NC (44.06 Ω) (Figure S18 Supporting Information), which confirmed the efficient charge dynamics of CuO/NC.

The ORR kinetics was further investigated by calculating the Tafel slope. In Figure 3c, CuO/NC demonstrated a Tafel slope of 53.08 mV dec^−1^, smaller than that of commercial Pt/C (93.28 mV dec^−1^) and Cu/NC (57.80 mV dec^−1^). As an effective parameter for evaluating the interfacial properties of the catalyst in the electrolyte, the electrochemical active specific surface area (ECSA) was determined by measuring the double‐layer capacitance (*C*_dl_). In Figure 3d and Figure S19 (Supporting Information), the calculated *C*_dl_ of CuO/NC (130.66 mF cm^−2^) is higher than Cu/NC (115.07 mF cm^−2^), suggesting that the increased exposure of CuO active species and the sufficient contact of O_2_ to active sites.^[^ ^59^ ^]^ The reaction kinetics of the electrode process was determined by measuring the polarization curves at different rotation speeds and according to the Koutecky–Levich equation (Figures S20, S21, Supporting Information). The electron transfer number (*n*) of CuO/NC is ≈3.8, which showed a higher efficiency of four‐electron process from O_2_ to OH^−^ than that of Cu/NC (n≈3.5). The hydrogen peroxide yield (H_2_O_2_%) was calculated according to the current recorded by the ring electrode of the RRDE. The H_2_O_2_% selectivity of CuO/NC is less than 4% with the *n* of over 3.92 in the potential range from 0.2 to 0.9 V (Figure 3e). These results are superior to Cu/NC and commercial Pt/C. To investigate the durability of the various catalysts, we performed chronoamperometric test. CuO/NC maintained the 91.3% of the normalized current after 50 h test at 0.6 V (Figure 3f). In contrast, the Cu/NC and commercial Pt/C catalyst decreased to only 80.5% after 50 h or 76.4% after about 15 h. The robustness of CuO/NC catalyst is further verified by a fast‐accelerated durability test (ADT). Figure S22 (Supporting Information) shows a negligible *E*_1/2_ loss after 50000 cycles (with only 30 mV drop). After the ADT test, TEM‐EDX images demonstrate the nearly unchanged octahedral geometry of the carbon support and the crystal structure (Figures S23, S24, Supporting Information) and the results of XRD and XPS (Figure S25, Supporting Information) also confirmed this conclusion. This once again confirmed the excellent durability of CuO/NC. The enhanced ORR performance of CuO/NC could be attributed to its high reactivity with O_2_. The non‐degeneracy of the 3d^9^ electronic structure of Cu in the coordination environment caused the splitting of the density of states. To the best of our knowledge, the performance of our fabricated CuO/NC catalyst surpasses most non‐precious metal electrocatalysts documented in the literature as summarized in Table S6 (Supporting Information).

**Figure 4:**
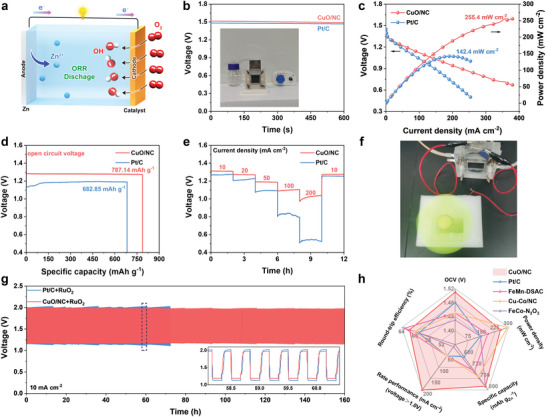
a) Depiction of the Zn–air battery. b) OCP curves of CuO/NC‐based ZAB and Pt/C‐RuO_2_‐based ZAB (inset: photograph of battery device). c) The power density plots from discharge polarization curves for corresponding ZABs. d) Specific capacities normalized by mass loss of polished Zn plate at 10 mA cm^−2^. e) Discharge curves of corresponding ZABs at various current densities from 10 to 200 mA cm^−2^. f) Photograph of a small fan running with the assistance of a Zn–air battery equipped with the CuO/NC catalyst. g) Galvanostatic discharge/charge cyclic curves for corresponding ZABs at 10 mA cm^−2^. h) Comparison of OCV, Round‐trip efficiency, Rate performance, Specific capacity, and Power density for CuO/NC, Pt/C, FeMn‐DSAC, Cu‐Co/NC., and FeCo‐N_3_O_3_‐based ZABs.

Based on the excellent ORR performance of CuO/NC, we assemble a primary Zn–air battery (ZAB) with CuO/NC loaded on carbon paper as an air cathode, a zinc plate as an anode, and 6 m KOH aqueous solution as an electrolyte (**Figure** **4**a). The alkaline ZAB catalyzed by CuO/NC exhibits a stable and exceptional open‐circuit potential of up to 1.51 V (Figure 4b). A peak‐power density of 255.4 mW cm^−2^ is achieved (Figure 4c), superior to that of commercial Pt/C catalyst (1.48 V, 142.4 mW cm^−2^) under the identical conditions. In addition, the specific capacity of CuO/NC‐based ZAB is estimated to be 787.14 mAh g_Zn_^−1^ at a current density of 10 mA cm^−2^ (Figure 4d), which is higher than Pt/C‐based ZAB (682.85 mAh g_Zn_^−1^). In Figure 4e, galvanostatic discharge curves display the rate performance of CuO/NC‐based ZAB at the current densities from 10 to 200 mA cm^−2^. Under the discharge condition at a high current density (200 mA cm^−2^), the CuO‐based ZAB maintained a discharge voltage of over 1.0 V, whereas the Pt/C‐based ZAB only reached ≈0.5 V. Moreover, the discharge potential recovered to 1.3 V when the current density switching back to 10 mA cm^−2^. This indicated a good rate of performance and reversibility of CuO/NC‐based ZAB. We then assembled the rechargeable ZAB by using CuO/NC as the cathode catalyst and RuO_2_ as the cathode catalyst with a mass ratio of 1:1. The cycling performance was investigated by galvanostatic discharge‐charge cycles at a current density of 10 mA cm^−2^. Figure 4f shows an electric fan powered by CuO/NC‐based ZAB, demonstrating the feasible application of CuO/NC in practical devices. Compared to Pt/C+RuO_2_‐based ZAB, CuO/NC+RuO_2_‐based ZAB shows negligible voltage decay within 160 h (Figure 4g), confirming the outstanding stability as aforementioned. In addition, a high and robust round‐trip efficiency was also demonstrated in CuO/NC+RuO_2_‐based ZAB, yielding 63.25% and still retaining 60.31% after 400 cycles (Figure S26, Supporting Information). More importantly, the CuO/NC‐based ZAB exhibited a superior performance than other benchmarking counterparts in terms of peak power density, specific capacity, and cycling stability (Figure 4h; Table S7, Supporting Information).^[^ ^60^, ^61^, ^62^ ^]^

**Figure 5:**
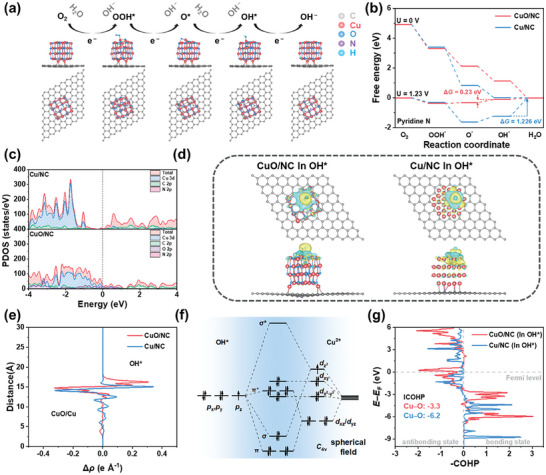
a) The ORR reaction pathway and the structure of adsorption intermediates for the CuO/NC. b) The calculated Gibbs free‐energy diagrams of ORR on Cu/NC and CuO/NC. c) The projected density of states (PDOS) of Cu/NC and CuO/NC. d) The charge density difference plots of Cu/NC and CuO/NC: the deletion and accumulation of electrons are represented by the blue and yellow contours, respectively. (The iso‐surface corresponds to 0.0005 e Å^−3^.) e) Planar‐average charge density analysis of CuO/NC and Cu/NC with OH^*^ adsorbed, respectively. f) d‐p orbital hybridization between the O atom in OH^*^ and Cu atom of the CuO cluster in the CuO/NC model. g) COHP analysis for OH adsorbed in Cu/NC and CuO/NC models.

To gain an in‐depth understanding of the superior ORR performance in CuO/NC‐based systems, the spin‐polarized DFT calculations were conducted. The ORR reaction pathway and adsorption intermediates, including OOH^*^, O^*^, and OH^*^ on the pyridinic nitrogen doped graphene‐supported CuO (CuO/NC) are illustrated in **Figure** **5**a and Figures S27–S29 (Supporting Information). The Cu/NC and CuO/NC exhibit downhill free‐energy pathways at U = 0 V vs. RHE (Figures 5b; Figures S30, S31, Supporting Information). It indicates that all the elementary ORR steps are spontaneous exothermic process and high ORR catalytic activity for both Cu/NC and CuO/NC systems. Upon increasing the potential to 1.23 V, for pyridinic nitrogen‐doped graphene, the rate limiting step for Cu/NC and CuO/NC are the third OH^*^ adsorption and the final OH^−^ desorption step, respectively. The CuO/NC structure has a significantly lower overpotential (0.230 V) than that of Cu/NC structure (1.226 V), suggesting a superior ORR activity for CuO/NC. Compared to Cu/NC systems, CuO/NC displays a significantly higher density of states of Cu *d* orbital at the Fermi level (Figure 5c; Table S4, Supporting Information). This could lead to the superior ORR catalytic activity performance of CuO/NC.^[^ ^63^, ^64^ ^]^ Bader charges analysis for OH* intermediates (Figure 5d; Figure S32, and Table S5, Supporting Information) reveals a more electron transfer on Cu/NC (0.61 e for OH^*^), consistent with the strong adsorption of OH^−^ on the three‐fold Cu hollow site than the weak adsorption of OH^−^ on the Cu top site of CuO/NC (0.46 e for OH^*^). Compared with the Cu/NC system, less electrons are obtained by OH groups in the OH^*^ intermediates of CuO/NC, resulting in substantial weaker binding energies for OH^*^. This is useful for the rate‐limiting step of OH^−^ desorption.^[^ ^13^, ^56^ ^]^ As shown in Figure 5e, the plane‐averaged (*X–Y*‐plane) charge density calculation along the Z direction (perpendicular to the interface) shows the larger charge density difference (Δ*ρ*) on the OH^*^ side, which suggests a more significant charge transfer between Cu and O atoms, corresponding to a more robust adsorption of the OH^*^ intermediate.

To achieve a deeper insight into the moderate strength adsorption of OH groups on the CuO/NC system, the schematic diagram of *d‐p* orbital hybridization is plotted in Figure 5f, consisting of O atom in OH and Cu atom of CuO cluster in CuO/NC model. The Cu atoms on the surface of CuO nanoclusters are in the coordinated environment with *C*_4v_ symmetry, where the *d* orbitals are splitting into $d_{z^{2}}$, *d_xy_*, $d_{x^{2}-y^{2}}$, and two‐fold degenerate orbitals *d*_*xz*/*yz*_. When OH group adsorbing on CuO/NC, the $d_{z^{2}}$ orbitals formed σ and σ^*^ bond with *p_z_* orbitals of hydroxyl oxygen while the other *d* orbitals form π and π^*^ bonds with *p*_*x*/*y*_ orbitals of hydroxyl oxygen and non‐bonding orbitals. Due to the 3d^9^ electronic configuration of Cu^2+^, the π and π^*^ bonds and non‐bonding orbitals are fully filled. This indicates that only the σ bonds contribute to the effective bonding in the OH^*^ and benefit to form a moderate strength adsorption to lower the activation energy barrier.^[^ ^58^ ^]^ Furthermore, the corresponding integral of crystal orbital Hamilton population (ICOHP) in Figure 5g for the Cu─O bonds of OH^*^ intermediates shows a decreasing negative value,^[^ ^65^ ^]^ changing from −6.2 eV in Cu/NC to −3.3 eV in CuO/NC, indicating a weaker interaction between Cu and O of OH^*^ intermediates in CuO/NC. This could be the reason of the enhanced OH adsorption free‐energy.^[^ ^66^, ^67^ ^]^ The theoretical results are in good agreement with the superior overpotential of CuO/NC.

## 3 Conclusion

In summary, we synthesized the nitrogen‐doped porous carbon‐supported ultra‐small CuO nanoparticles under γ‐ray irradiation induction. The as‐prepared catalyst shows an extraordinary ORR activity in alkaline electrolytes and exhibits an advanced performance when used as a ZABs electrode. Both experimental and theoretical studies demonstrated that the Cu^2+^ on the surface of CuO (111) crystal plane alongside *C*_4v_ coordination configuration by constructing CuO NPs with broken symmetry, leading to the separation of paired electrons in *d* orbitals. This facilitates the charge transfer localized on the OH^*^ rather than the Cu─O bond when it binds to the key intermediate OH^*^, thereby lowering the energy barrier for OH^*^ desorption. The reduction enhances the catalytic activity of CuO/NC in the ORR process and thus improves the performance of ZABs. Our study provides an innovative avenue for the practical commercialization of the CuO/NC catalysts.

## Conflict of Interest

The authors declare no conflict of interest.

## Supporting information



Supporting Information

## Data Availability

The data that support the findings of this study are available from the corresponding author upon reasonable request.
