# Supplementary material for: Symmetry Breaking in Rationally Designed Copper Oxide Electrocatalyst Boosts the Oxygen Reduction Reaction
Source: Adv Sci (Weinh). 2024 Dec 16;12(6):2411928. doi: 10.1002/advs.202411928 (PMC11809328; doi:10.1002/advs.202411928)
Supplement: Supplementary file 1 — Supporting Information [file ADVS-12-2411928-s001.docx]

**Supporting Information**

**Symmetry Breaking in Rationally Designed Copper Oxide Electrocatalyst Boosts the Oxygen Reduction Reaction**

*Haoyu Peng*^+^, *Weiyi Wang*^+^, *Jiyuan Gao*, *Fan Jiang*, *Bowei Li,* *Yicheng Wang*, Yiqian Wu, *Yue Wang**, *Jiuqiang Li*, *Jing Peng*, *Wei Hu*, *Zhenhai Wen, Dingsheng Wang*, *Erhuan Zhang**, and *Maolin Zhai**

**Supporting Information**

**Table of Contents**

**Chemicals and reagents (Page 1)**

**Synthesis procedure (Page 1–2)**

**Material characterizations (Page 2–3)**

**Electrochemical measurements (Page 3–5)**

**Zn-air battery measurements (Page 5)**

**DFT Calculation (Page 5–6)**

**Supporting Figures (Page 7–21)**

**Supporting Tables (Page 22–28)**

**Experiment section**

**Chemicals and reagents**

Zinc chloride (ZnCl_2_, Beijing Mairuida Technology Co., Ltd., AR), cupric acetate (Cu(COOH)_2_·H_2_O, J&K Scientific, ≥98.5%), cupric acetate monohydrate (CuCl_2_·2H_2_O, Beijing Tongguang Fine Chemical Co., AR), 1*H*−1,2,3-Triazol (Shanghai Dibo Biotechnology Co., Ltd., ≥98%), ammonium hydroxide (Beijing Lanyi Chemical Products Co., Ltd., 25%), N, N-dimethylformamide (DMF, Tianjin Concord Technology Co., Ltd., CP), methanol (MeOH, Beijing Tongguang Fine Chemical Co., AR), isopropyl alcohol (Beijing Tongguang Fine Chemical Co., AR), Nafion perfluorinated resin solution (Shanghai Hesen Electric Co., Ltd., 5%), 20 wt% Pt/C catalysts (Suzhou Shengernuo Technology Co., Ltd.). All chemicals were of analytical grade and used without further purification.

**Synthesis procedure**

**Synthesis of MET−6 and NC-900**

The MET−6 MOF was prepared by a one-pot method at room temperature according to a previously reported method.^[1]^ Typically, 1.00 g of ZnCl_2_ was dissolved in 14 mL of water and stirred for 5 min. 10 ml of MeOH and DMF were added into the ZnCl_2_ solution, respectively. Ammonium hydroxide was added dropwise into the mixture solution with stirring for 5 min to form a homogeneous solution. 0.94 mL of 1*H*−1,2,3-Triazol was dropwise added to this solution under stirring at room temperature to form a precipitate and reacted for 12 hours at room temperature. The solid was collected by centrifugation, washed with MeOH three times and dried under oven at 60 °C for 12 h.

300 mg of MET−6 was placed in a quartz boat and then heated at 900 °C for 1 h in a tube furnace with a heating rate of 5 °C min^−1^ under flowing Ar atmosphere. After cooling to room temperature, the nitrogen-doped carbon (NC-900) was obtained.

**Radiation Synthesis of CuO/NC**

10 mg of NC-900 and 1.6 mg of Cu(COOH)_2_·H_2_O were dispersed into 10 mL water. And then, 10 ml of isopropyl alcohol was added into the solution with sonicated for 8 h. ​The mixture solution was continuously aerated with N_2_ for 15 min before sealed. The sample was then irradiated using a ^60^Co γ source with 50,000 curies for 50 min (photon energies of 1.33 and 1.17 MeV was used for gamma irradiation, Institute of Applied Chemistry, Peking University). The dose rate was approximately 100 Gy min^−1^, as traced by a Fricke dosimeter. The as-prepared product was collected by centrifugation, washed with water three times and dried under oven at 60 °C for 12 h.

**Radiation Synthesis of Cu/NC**

10 mg of NC-900 and 3.2 mg of CuCl_2_·2H_2_O were dispersed into 20 ml of a mixed solution of water and isopropanol (V_water_/V _isopropanol_=1/1) with sonicated for 8 h. After protected by N_2_ and sealed, the sample was then irradiated using a ^60^Co γ source with 100 min. The dose rate was approximately 100 Gy min^−1^. The as-prepared product was collected by centrifugation, washed with water three times and dried under oven at 60 °C for 12 h.

**Material characterizations**

Powder X-ray diffraction (PXRD, X-Pert3 Power) pattern were characterized with Cu Ka radiation (λ = 0.154056 nm) in the range of 5−60°. SEM images were captured using a scanning electron microscope (SEM, Hitachi S-4800) at an acceleration voltage of 5 kV. The morphology, structure, and STEM-EDS elemental imaging of the material were observed by a field-emission transmission electron microscope (TEM, JEM−2100F) with an accelerating voltage of 200 kV and a filament of thermal field-emission lanthanum hexaboride. A micro-Raman imaging spectrometer (Raman, DXRxi) was used for characterization, with a laser wavelength of 532 nm and an output power of 3 mW. The chemical composition of samples was determined by X-ray photoelectron spectroscopy (XPS, AXIS Supra) with an exciting source of Al Kα (1286.6 eV). N_2_ sorption isotherms were measured at 77 K on Kubo X1000 sorption analyzer. The pore size distributions were calculated using the non-local density functional theory (NLDFT) and de-regularized Monte Carlo simulation method (GCMC). All samples were pre-activated under vacuum at 150 °C for 12 h before nitrogen sorption measurements. The X-ray absorption find structure spectra Cu K-edge were collected at BL14W beamline of Shanghai Synchrotron Radiation Facility (SSRF). The energy calibration for the present XANES/EXAFS spectra was done by simultaneously measuring the reference spectrum of Cu metal foil. All the measured XANES/EXAFS spectra were collected at room temperature in a transmission mode using gas-ionization detectors.

**XAFS and EXELFS Analysis**

The obtained XAFS data was processed in Athena (version 0.9.26) for background, pre-edge line and post-edge line calibrations. Then Fourier transformed fitting was carried out in Artemis (version 0.9.26). The k^2^ weighting, k-range of 3-14 Å^−1^ and R range of 1- ~3 Å were used for the fitting of Cu foil; k-range of 3-11.2 Å^−1^ and R range of 1- ~3 Å were used for the fitting of samples. The four parameters, coordination number, bond length, Debye-Waller factor and E_0_ shift (CN, R, ΔE_0_) were fitted without anyone was fixed, the σ^2^ was set.

For Wavelet Transform analysis, the χ(k) exported from Athena was imported into the Hama Fortran code. The parameters were listed as follow: R range, 1 - 4 Å, k range, 0 - 15 Å^−1^ for samples; k weight, 2; and Morlet function with κ=10, σ=1 was used as the mother wavelet to provide the overall distribution.

**Electrochemical measurements**

Electrochemical measurements were carried out using an electrochemical workstation (AUTOLAB PGSTAT302, Metrohm Autolab) coupled with a rotating-ring disk electrode (RRDE, IPS China Limited. The glassy carbon disk electrode with a diameter of 5.0 mm) in a four-electrode cell, in which Ag/AgCl (saturated KCl solution) and graphite rod was used as reference and counter electrodes, respectively. The catalyst ink was prepared by ultrasonically dispersing 4.0 mg catalysts in 0.4 mL of a mixture containing water (280 μL), isopropanol (100 μL) and Nafion (5 wt%, 20 μL). The suspension was sonicated to form a homogeneous suspension and 10 μL of suspension was dropped onto the surface of the pre-polished glassy carbon electrode to achieve a mass loading of 0.5 mg_cat_. cm^−2^ (commercial Pt/C, 0.1 mg_Pt_ cm^−2^), leading to a uniform catalyst film. The electrolyte (0.1 M KOH) was saturated with Ar or O_2_ before test. The CV curves were recorded in O_2_ and Ar-saturated environment at a scan rate of 100 mV s^−1^, while the LSV curves were conducted using a RRDE in an O_2_-saturated environment at different rotating rates from 400 to 3600 rpm at a scan rate of 5 mV s^−1^. The long-term durability was performed through continuously cycling between 0.6 and 1.0 V (vs. RHE) in O_2_-saturated 0.1 M KOH at a scan rate of 100 mV s^−1^ for 50,000 cycles. The stabilities of catalyst samples and Pt/C were evaluated in O_2_ saturated electrolyte by chronoamperometry tests (0.6 V vs. RHE, 50 h). The double-layer capacitance (*C*_dl_) is used to estimate the electrochemically active surface area (ECSA) of the catalyst. It is obtained by testing the CV of the non-Faraday voltage range at different scan rates in 0.1 M KOH.

The number of electrons transferred (n) and kinetic current density (*J*_K_) were calculated according to the Koutecky-Levich equation:

$\frac{1}{j}=\frac{1}{j_{\text{L}}}+\frac{1}{j_{\text{K}}}=\frac{1}{0.62nFC_{0}D_{0}^{2/3}\nu^{-1/6}\omega^{1/2}}+\frac{1}{nFkC_{0}}$ (1)

where *J* is the measured current density, *J*_K_ and *J*_L_ are the kinetic and limiting current densities, *ω* is the angular velocity of the disk, n is the overall number of electrons transferred in oxygen reduction, *F* is the Faraday constant (96485 C mol^−1^), *C*_0_ is the bulk concentration of O_2_ (1.2 × 10^−6^ mol cm^−3^), *D*_0_ is the diffusion coefficient of O_2_ in 0.1 M KOH (1.9 × 10^−2^ cm^2^ s^−1^), and *V* is the kinematic viscosity of the electrolyte (0.01 cm^2^ s^−1^).

The yield of hydrogen peroxide (H_2_O_2_%) and the electron transfer number (n) were calculated by the followed equations:

$\text{H}_{2}\text{O}_{2}(\%)=\frac{2i_{r}}{N\left|i_{d}\right|+i_{r}}\times100\%$ (2)

$\text{n}=\frac{4\left|i_{d}\right|}{\left|i_{d}\right|+i_{r}/N}$ (3)

where *i*_D_ is the disk current, *i*_R_ is the ring current, and *N* is the ring collection efficiency and is determined to be 0.344.

**Zn-air battery measurements**

Primary ZABs were assembled by using polished zinc foil as the anode and 6 M KOH solution involving 0.2 M Zn(OAc)_2_ as the electrolyte. CuO/NC or commercial Pt/C coated on carbon paper was used as the air cathode. The total catalyst loading amount on carbon paper was 1.5 mg cm^−2^. The voltage-current polarization curves were obtained on an electrochemical workstation (CHI 760 E: CH Instrumental Inc.) with a scan rate of 10 mV s^−1^. Rechargeable ZABs were assembled by using aqueous solution containing 6 M KOH + 0.2 M Zn(OAc)_2_ as the electrolyte. CuO/NC and RuO_2_ (with a mass ration of 1:1) or 20% Pt/C and RuO_2_ (with a mass ration of 1:1) coated on carbon paper were used as the air cathode. The galvanostatic discharge/charge cycling stability for the ZABs were tested using a LANDCT2001A tester under ambient condition.

**DFT Calculation**

All the spin-polarized DFT calculations are performed by using the Vienna ab initio simulation package (VASP).^[2-3]^ A generalized gradient approximation is adopted based on the Perdew-Burke-Ernzerhof exchange-correlation functional and the effect of the van der Waals interactions is considered by the DFT-D3 method.^[4-5]^ The structure optimization and electronic property calculations are performed with Γ-centered 1×1×1 and 3×3×1 k-point grids. In addition, the vacuum regions are kept at least 15 Å apart along the c-axis to eliminate the effect of interlayer interactions. In all our calculations, the plane wave cutoff energy is set to 400 eV. The convergence criteria for the total energy and maximum force per atom are set as 10^-5^ eV and 0.04 eV Å^-1^, respectively.

For ORR in an alkaline electrolyte, the ORR pathway can be describe as follows:^[6]^

$\text{*O}_{2}\text{ + H}_{2}\text{O + e}^{\text{-}}\to\text{*OOH + OH}^{-}$ (4)

$\text{ *OOH + e}^{\text{-}}\to\text{O* + OH}^{-}$ (5)

$\text{O* + H}_{2}\text{O + e}^{\text{-}}\to\text{*OH + OH}^{-}$ (6)

$\text{*OH + e}^{\text{-}}\to\text{OH}^{-}\text{ + *}$ (7)

For each elementary step of ORR, the Gibbs reaction free energy (Δ*G*) is calculated as follows:

$\Delta G=\Delta E+\Delta E_{ZPE}-T\Delta S$ (8)

where $\Delta E$is the adsorption energy of each adsorbed intermediate, $\Delta E_{ZPE}$and $\Delta S$denotes the difference of zero point energy and entropy, respectively.

Three different types of graphene doped with nitrogen (graphitic, pyrrolic and pyridinic nitrogen doped graphene) loaded with CuO/Cu nanoparticles are investigated according to the experimental results. For Cu/NC-based system, the Cu_38_ cluster which has been widely used as a representative model for investigating the catalytic characteristics of copper nanoparticles, is used to decorate the three types of graphene doped with nitrogen surface in the calculations.^[7]^ Meanwhile, the CuO models contains 32 Cu atoms and 32 O atoms, respectively.

**Supporting Figures**


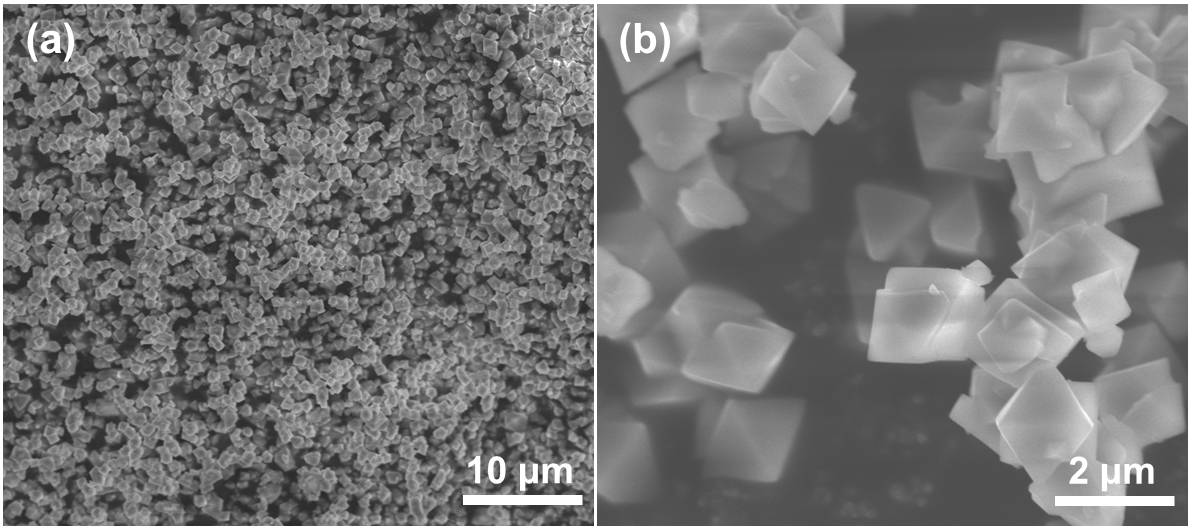


**Figure S1.** SEM images of MET-6.


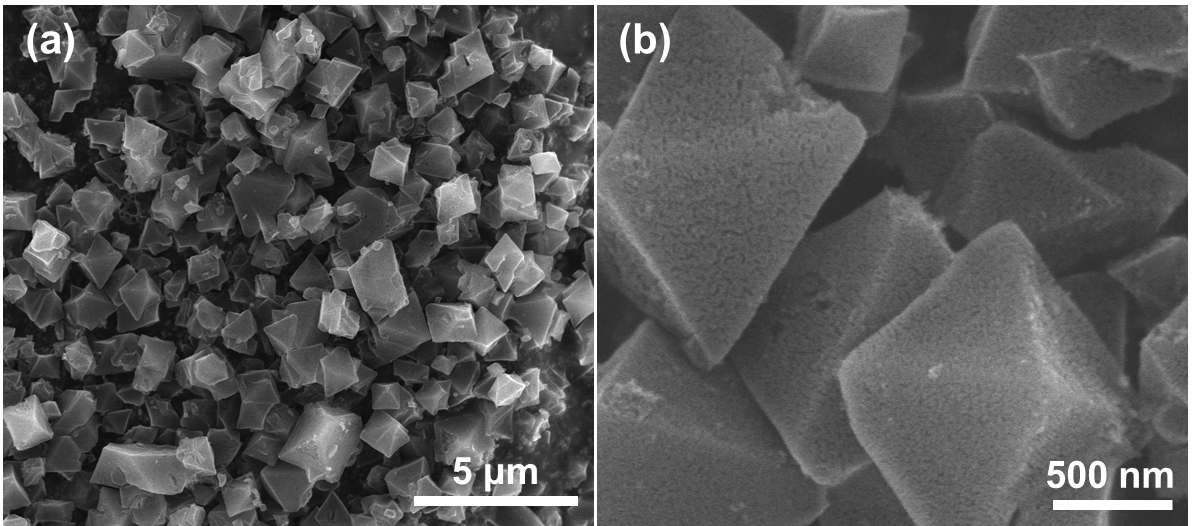


**Figure S2.** SEM images of NC-900.


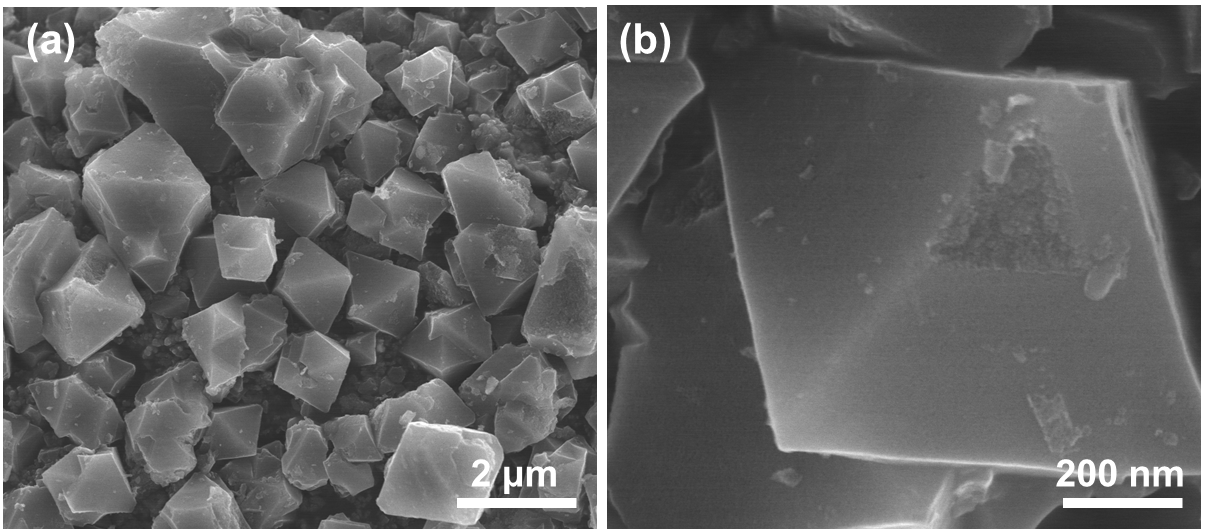


**Figure S3.** SEM images of CuO/NC.


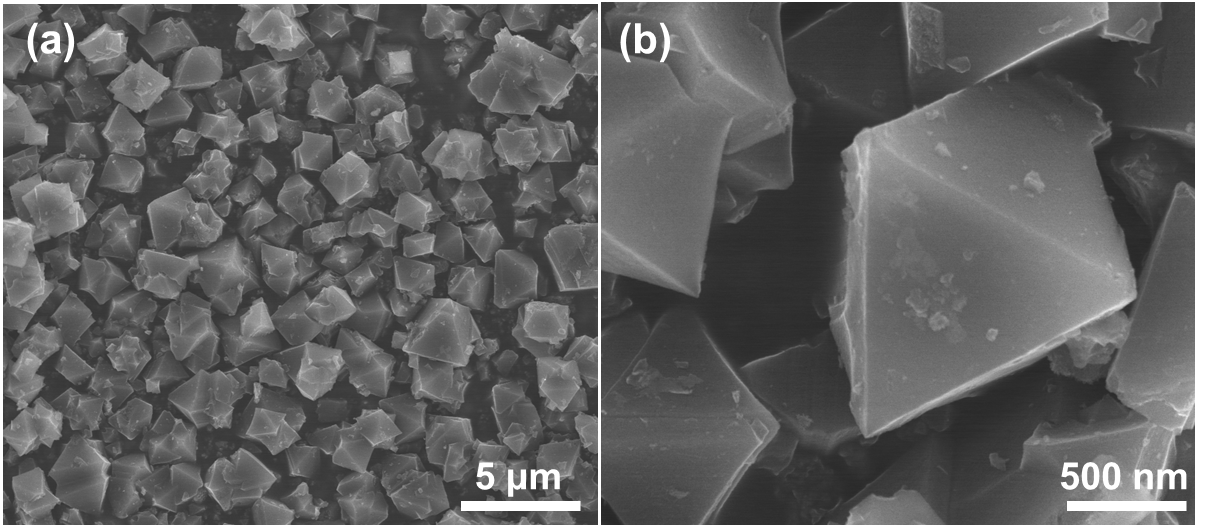


**Figure S4.** SEM images of Cu/NC.


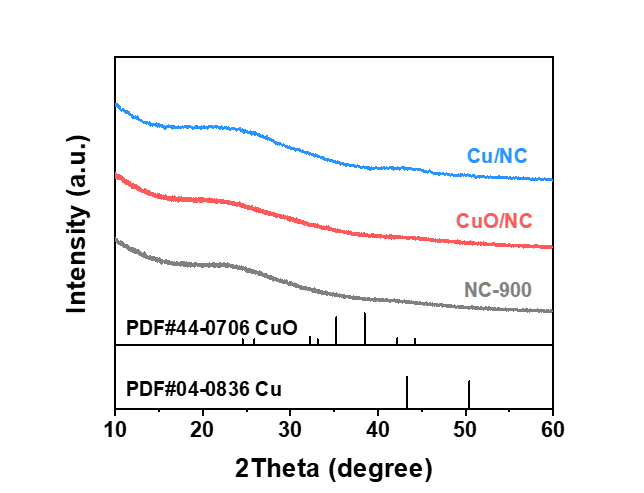


**Figure S5.** The PXRD patterns of NC-900, CuO/NC, and Cu/NC.

**Figure S6.** The Raman spectra for NC-900, CuO/NC, and Cu/NC.


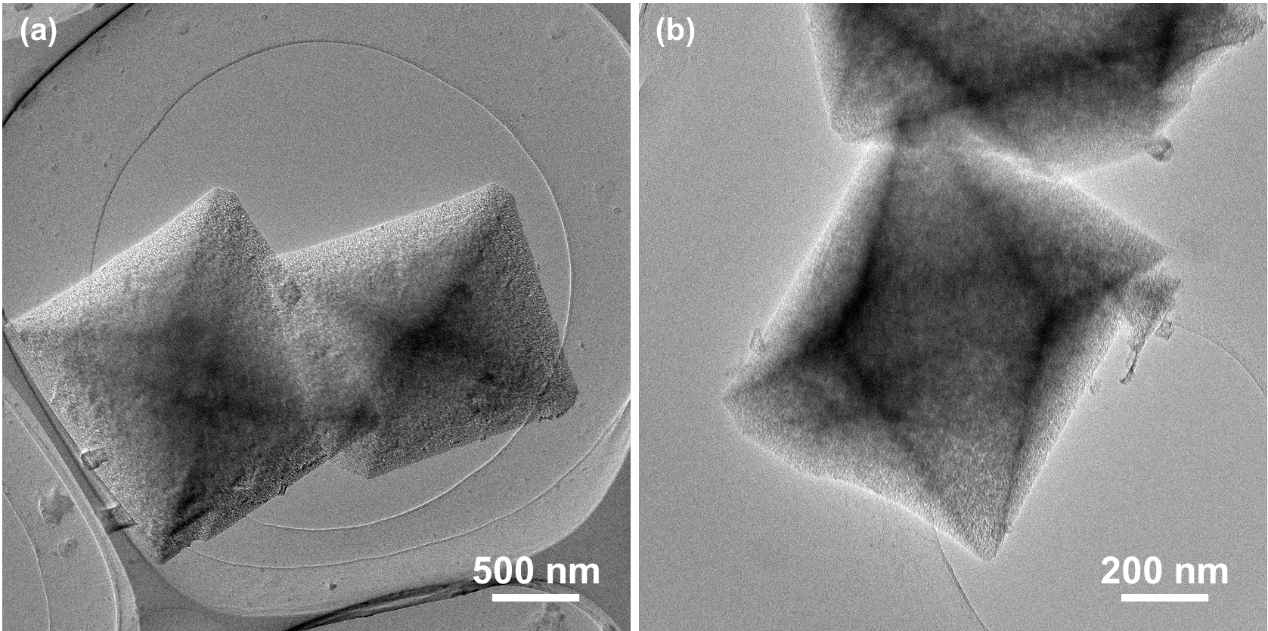


**Figure S7.** TEM images of (a) NC-900 and (b) CuO/NC.


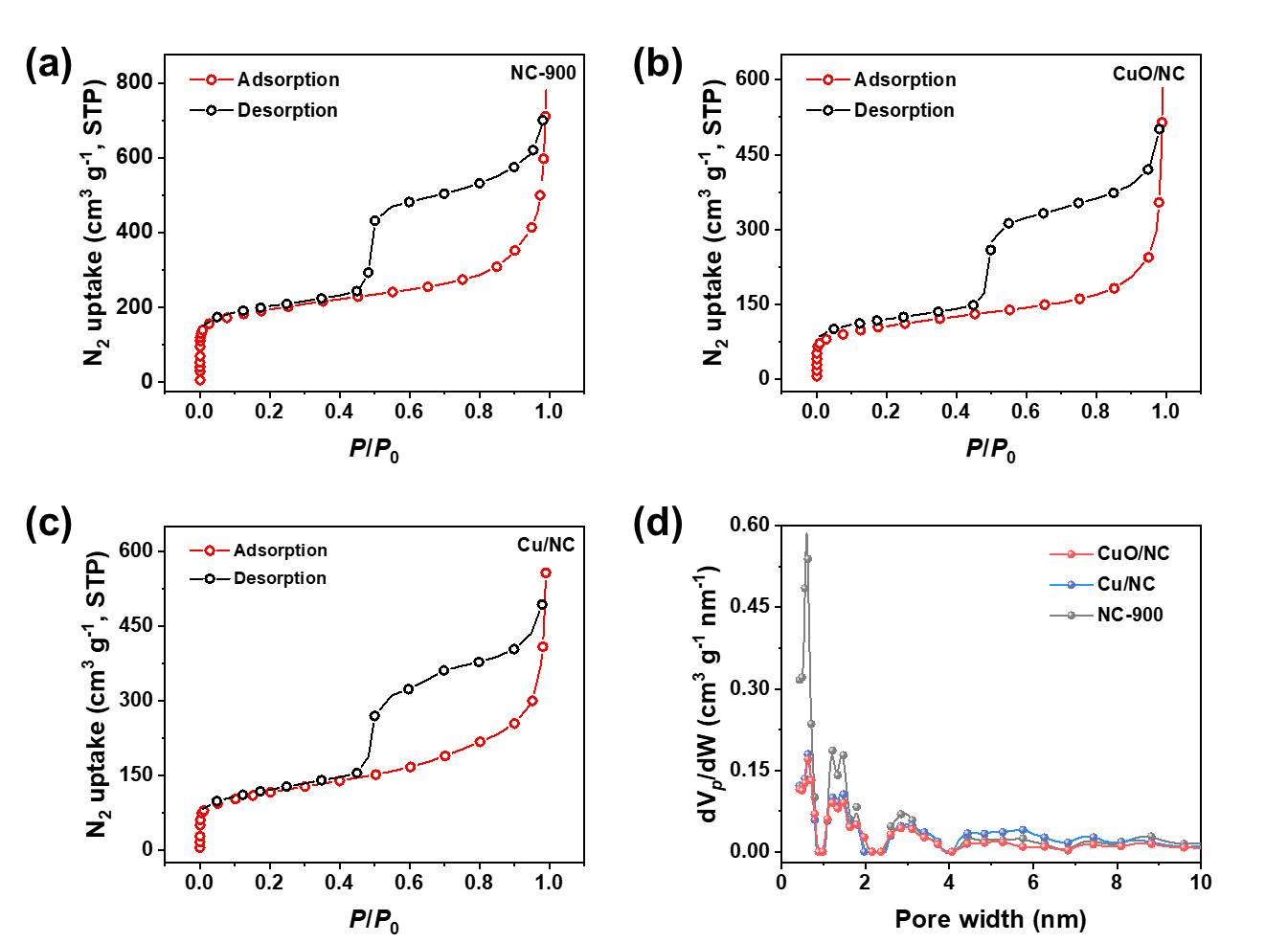


**Figure S8.** N_2_ adsorption isotherms of (a) NC-900, (b) CuO/NC, (c) Cu/NC at 77 K, and (d) their corresponding pore distribution.


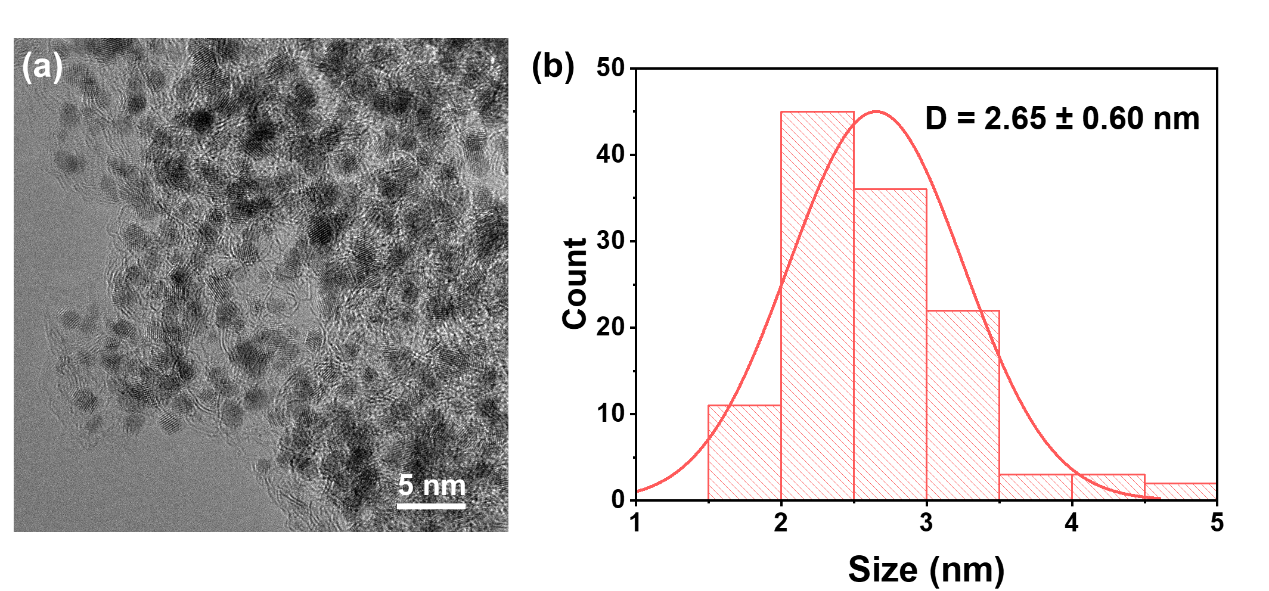


**Figure S9.** (a) HRTEM image of CuO/NC. (b) Particle size distribution of CuO NPs.


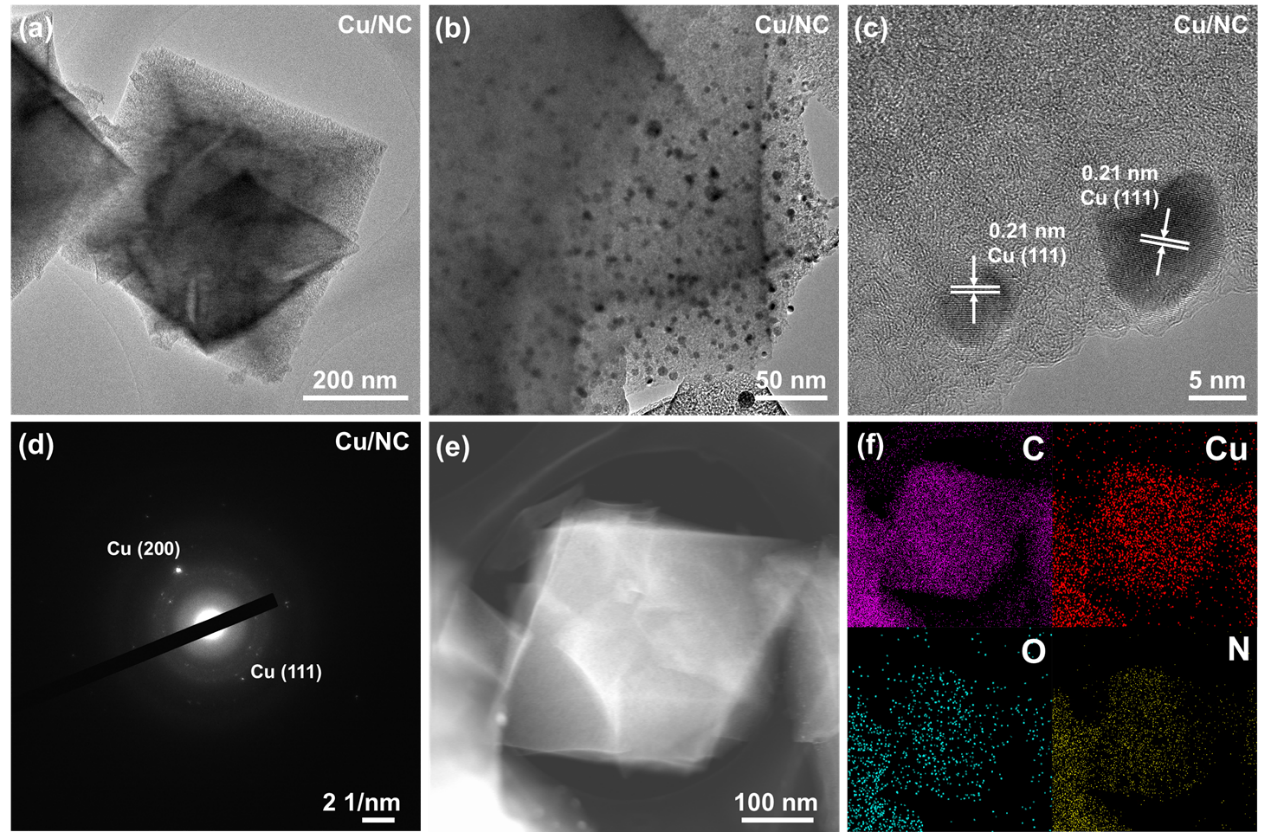


**Figure S10.** (a, b) TEM, (c) HRTEM, (d) SAED, (e) STEM images and (f) the corresponding EDX mappings of the resultant Cu/NC. (Oxygen mainly comes from the adsorption of oxygen in the air on the surface of material.)

**Figure S11.** XPS spectra of MET-6, NC-900, CuO/NC, and Cu/NC.


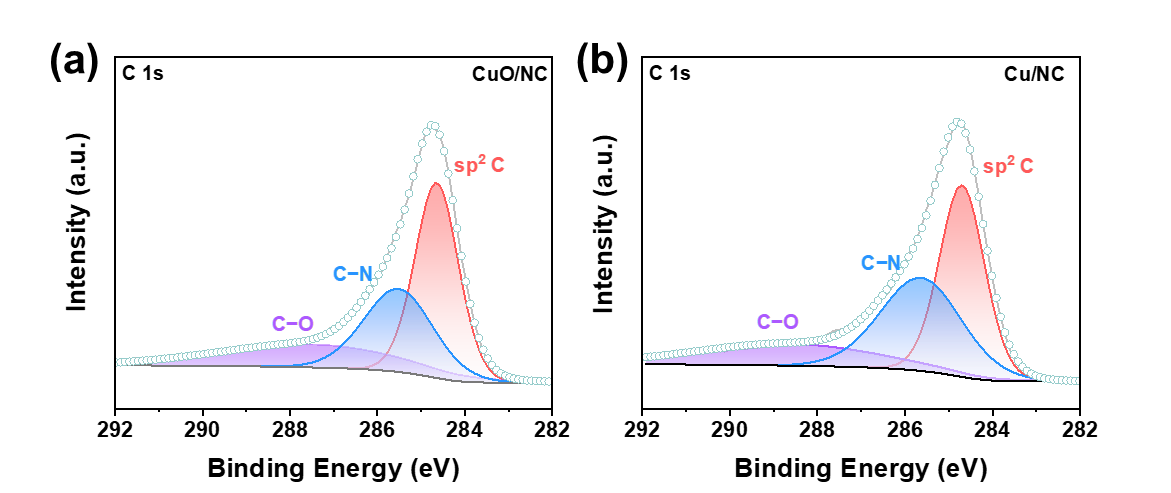


**Figure S12.** C 1s XPS spectra of (a) CuO/NC and (b) Cu/NC.


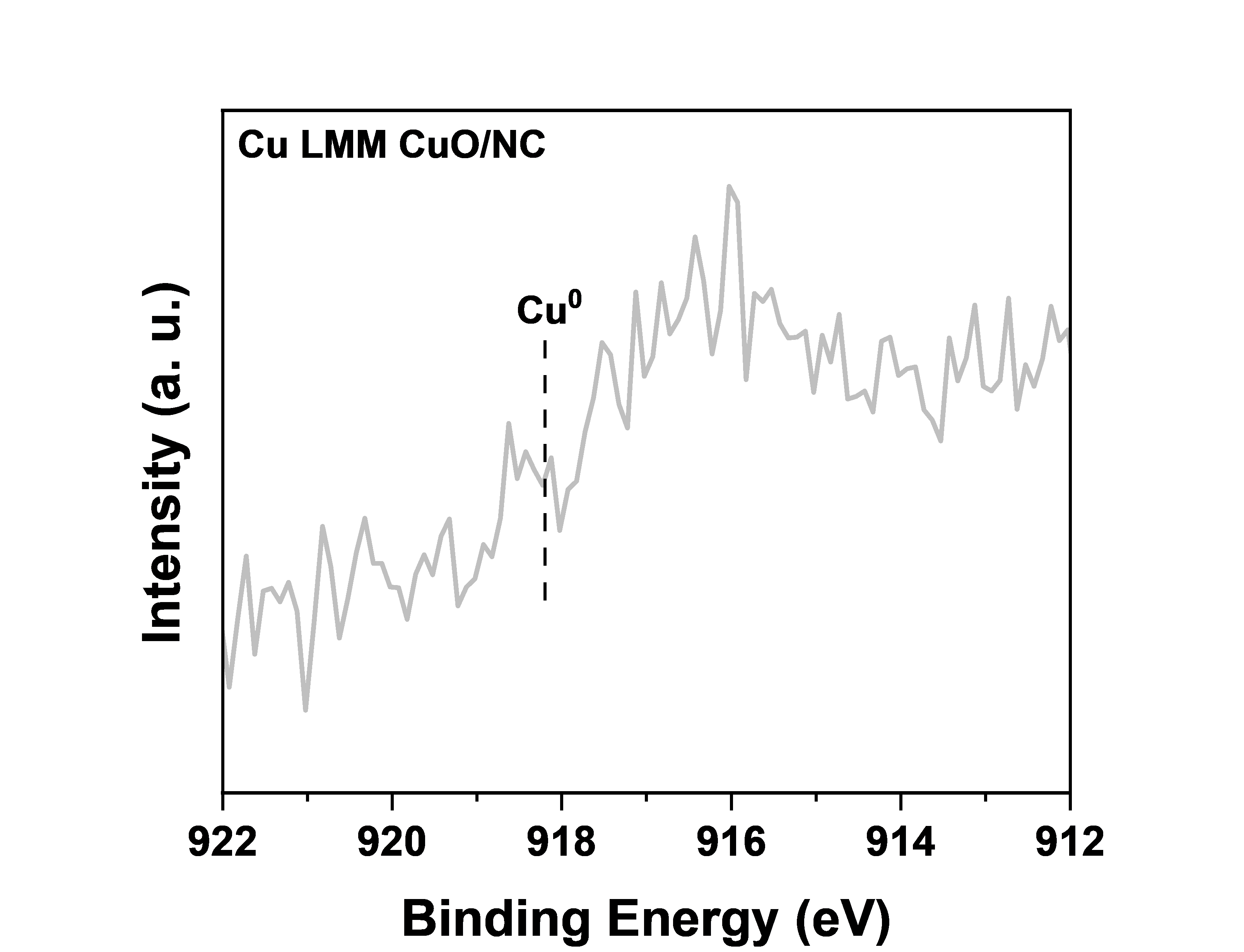


**Figure S13.** the Cu LMM spectrum of CuO/NC.


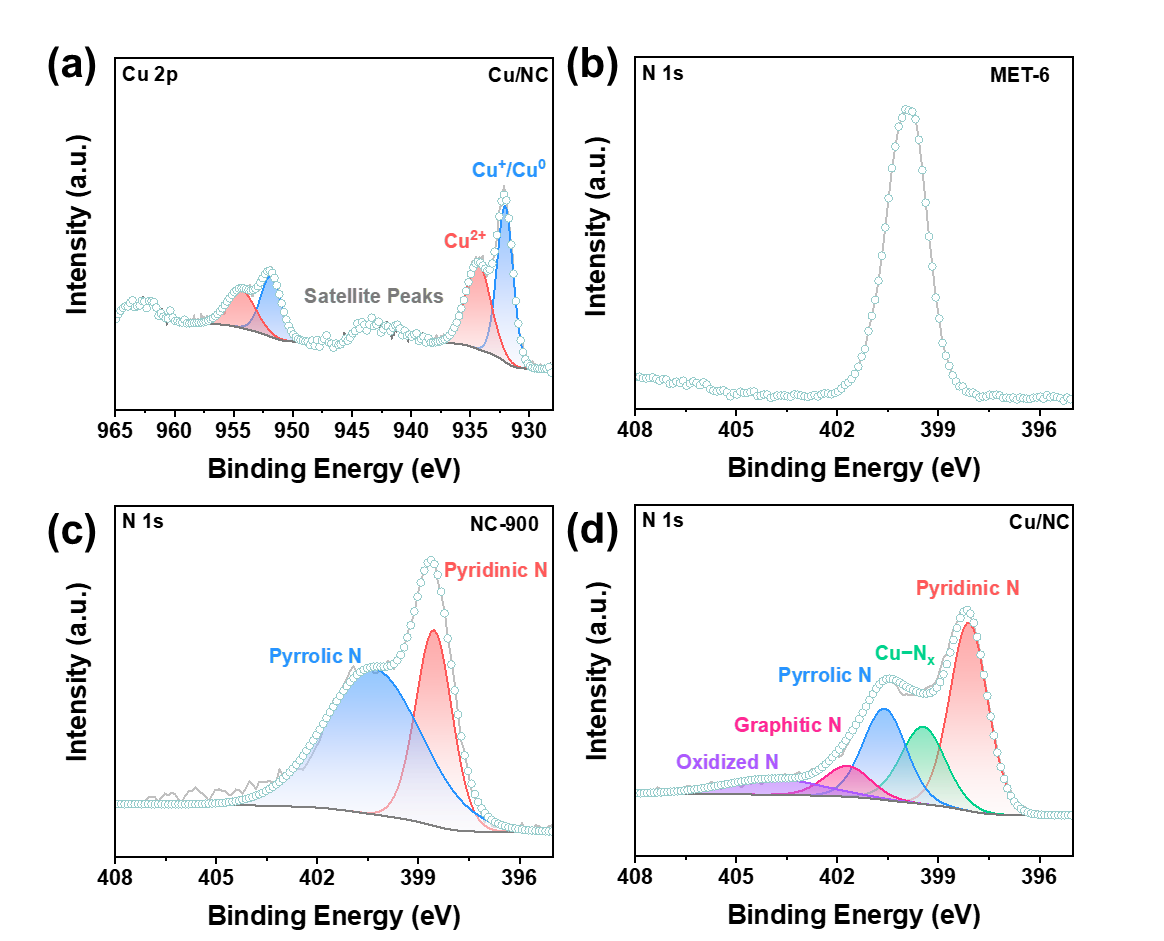


**Figure S14.** (a) Cu 2p XPS spectra of Cu/NC. N 1s XPS spectra of (b) MET-6, (c) NC-900, and (d) Cu/NC.

**Figure S15.** χ(R) space spectrum fitting curve of Cu K-edge for Cu foil.


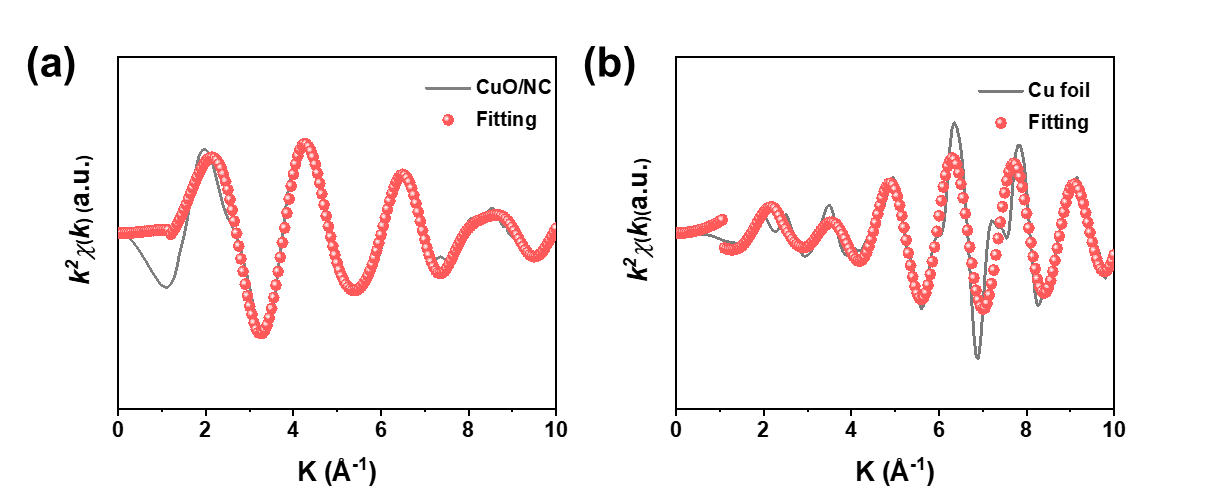


**Figure S16** The EXAFS analysis of (a) CuO/NC and (b) Cu foil in k space.


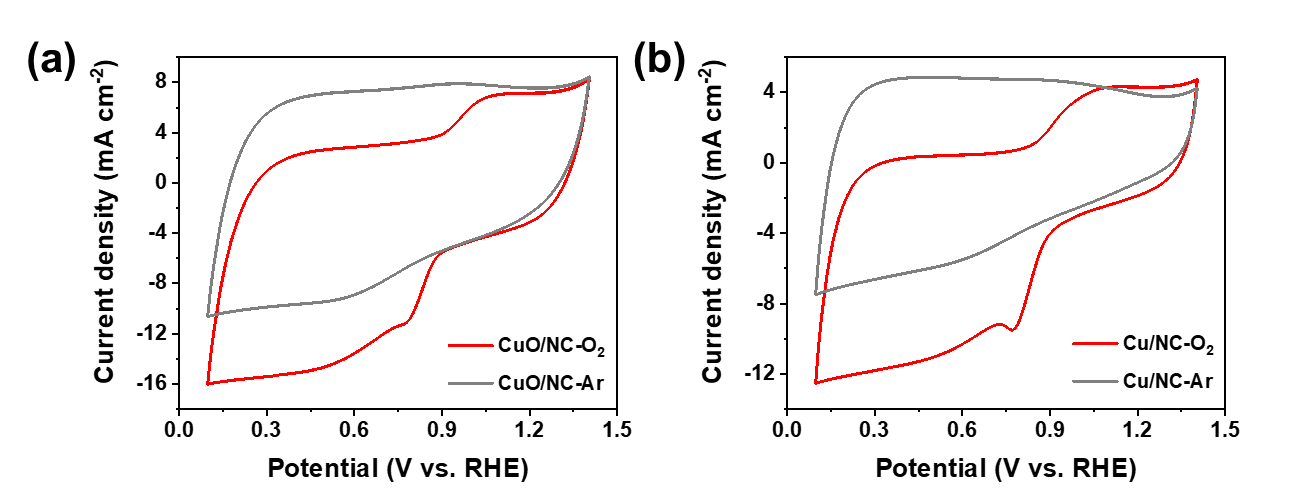


**Figure S17.** CV curves of (a) CuO/NC and (b) Cu/NC.


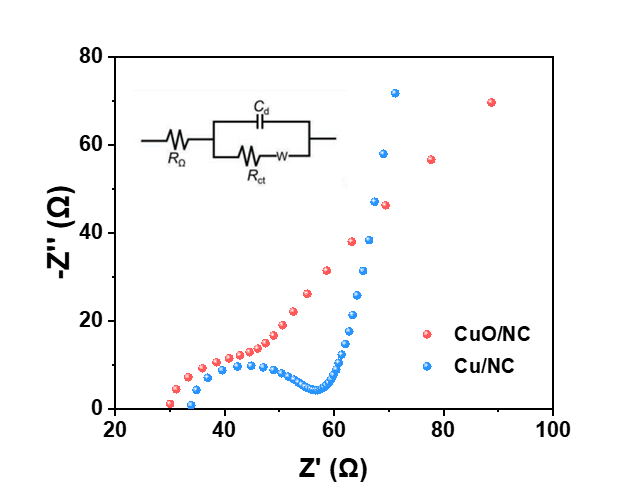


**Figure S18.** EIS plots of CuO/NC and Cu/NC.


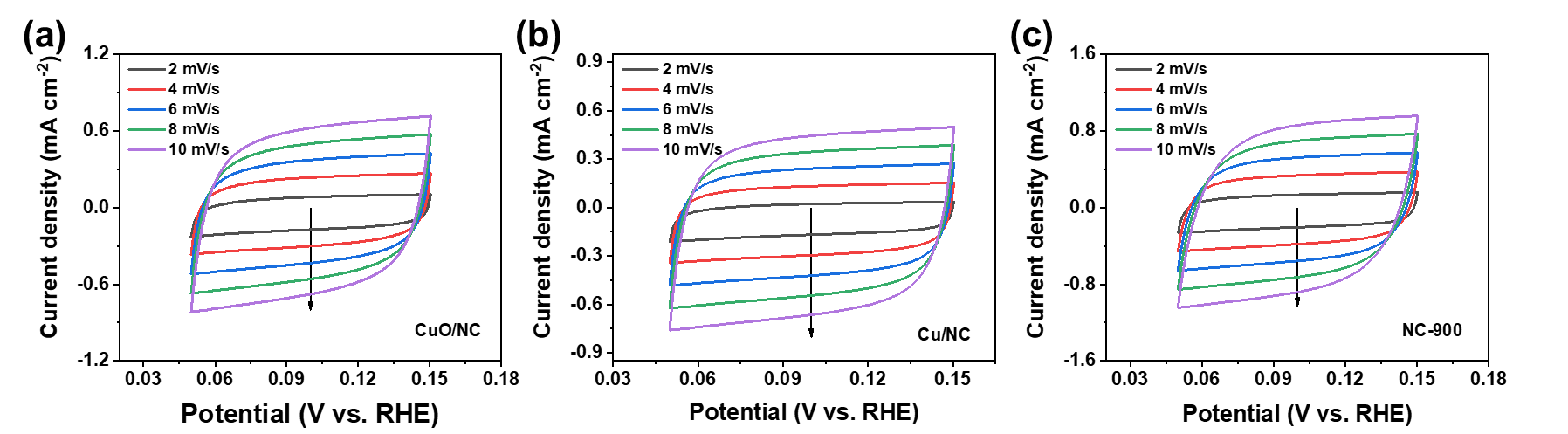


**Figure S19.** CV curves of (a) CuO/NC, (b) Cu/NC, and (c) NC-900 under 0.1 M KOH solution in the region of 0.05~0.15 V vs. RHE without Faradaic process.


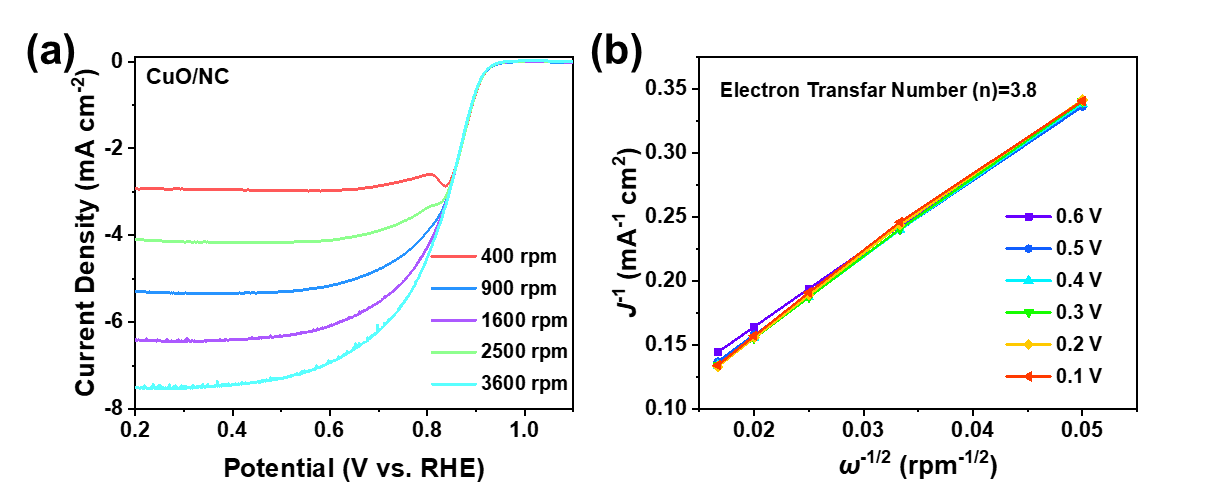


**Figure S20.** (a) The ORR polarization curves at different rotating rates and (b) K-L plots and electron-transfer number of CuO/NC.


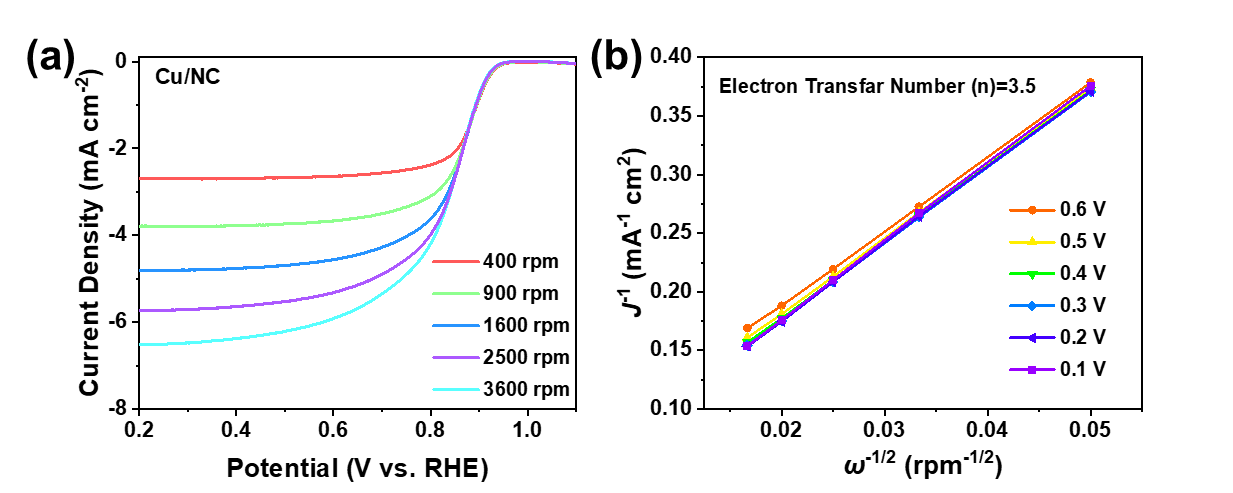


**Figure S21.** (a) The ORR polarization curves at different rotating rates and (b) K-L plots and electron-transfer number of Cu/NC.


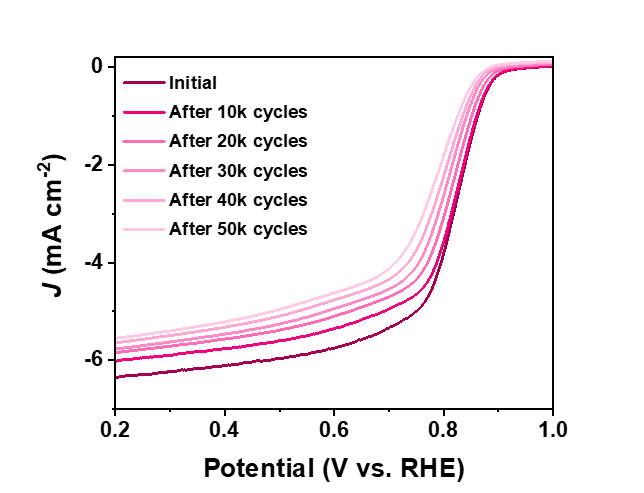


**Figure S22.** ORR polarization curves of CuO/NC before and after 10000, 20000, 30000, 40000 and 50000 cycles. The accelerated durability test was conducted by CV cycles (100 mV s^-1^) in O_2_-saturated 0.1 M KOH solution from 0.6 to 1.0 V vs. RHE during 50000 cycles.


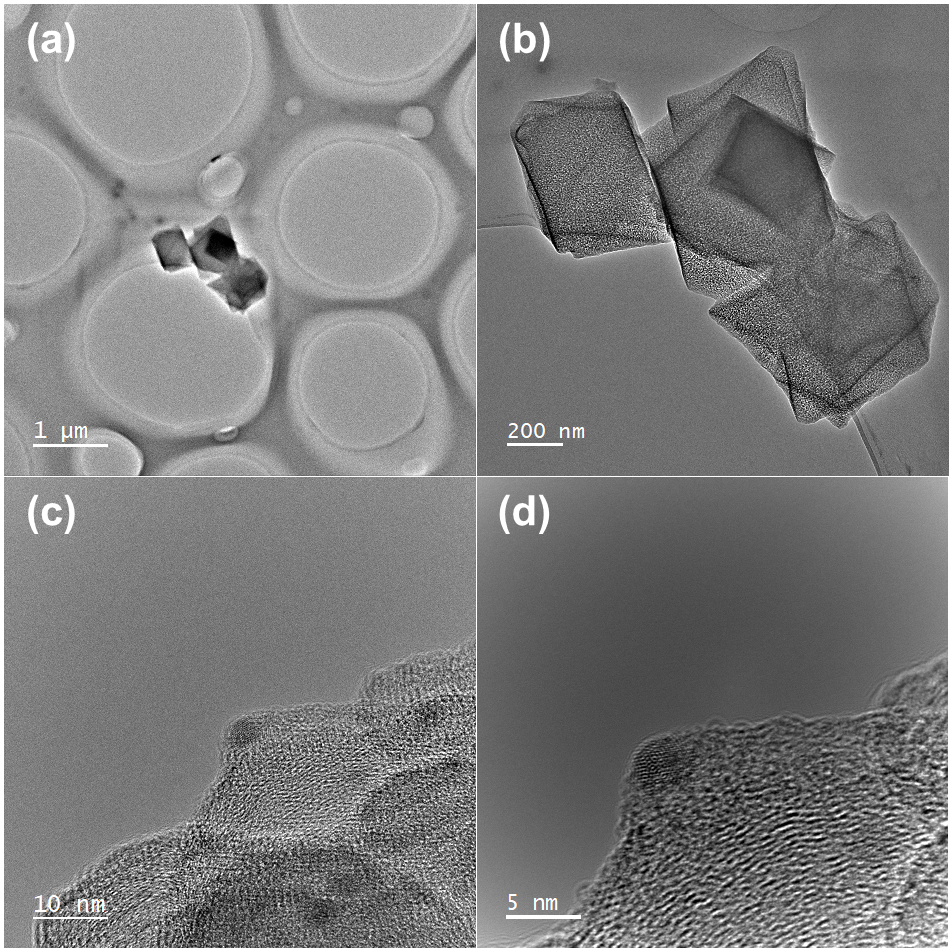


**Figure S23.** (a, b) TEM and (c, d) HRTEM images of CuO/NC after 50000 cycles.


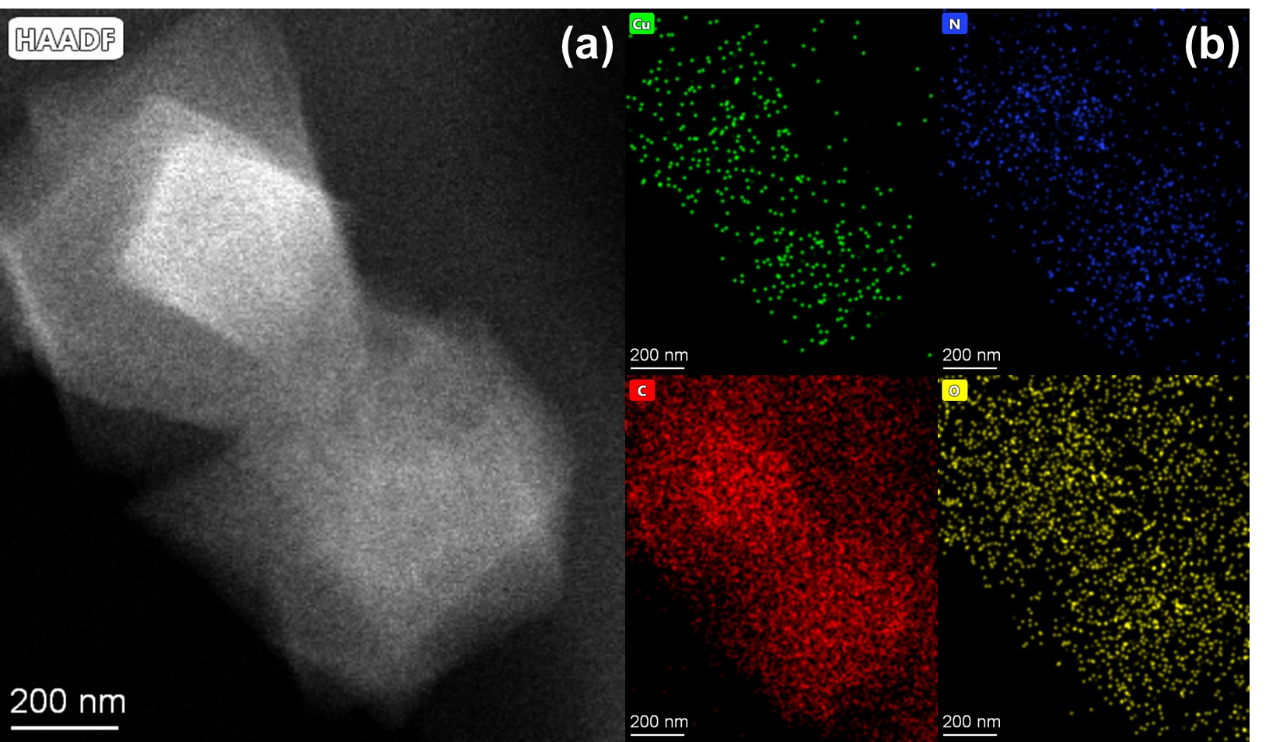


**Figure S24.** (a) HAADF-STEM image and (b) the corresponding EDX mappings of CuO/NC after 50000 cycles.


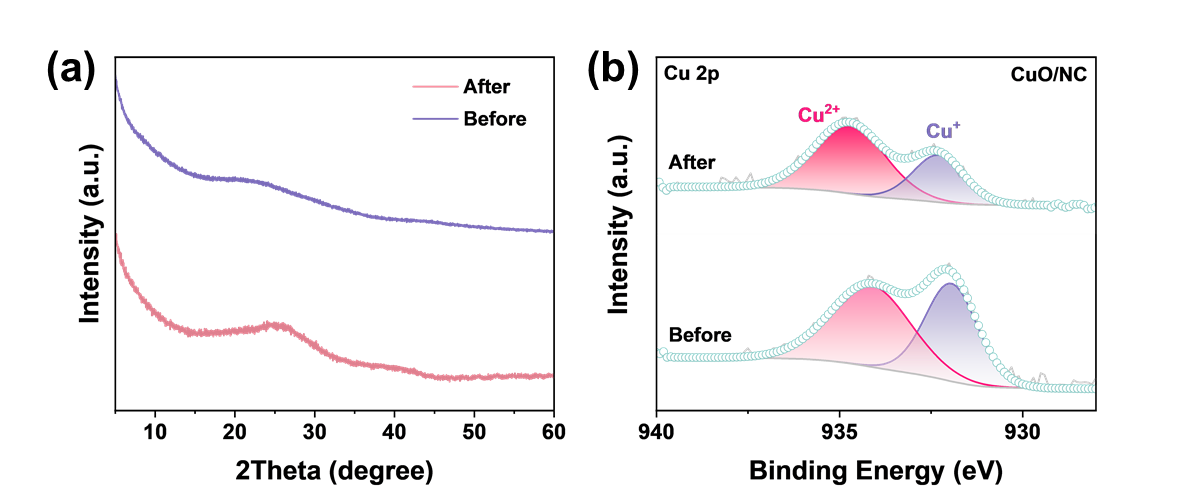


**Figure S25.** (a) XRD pattern and (b) Cu 2p XPS results of CuO/NC after 50000 cycles.


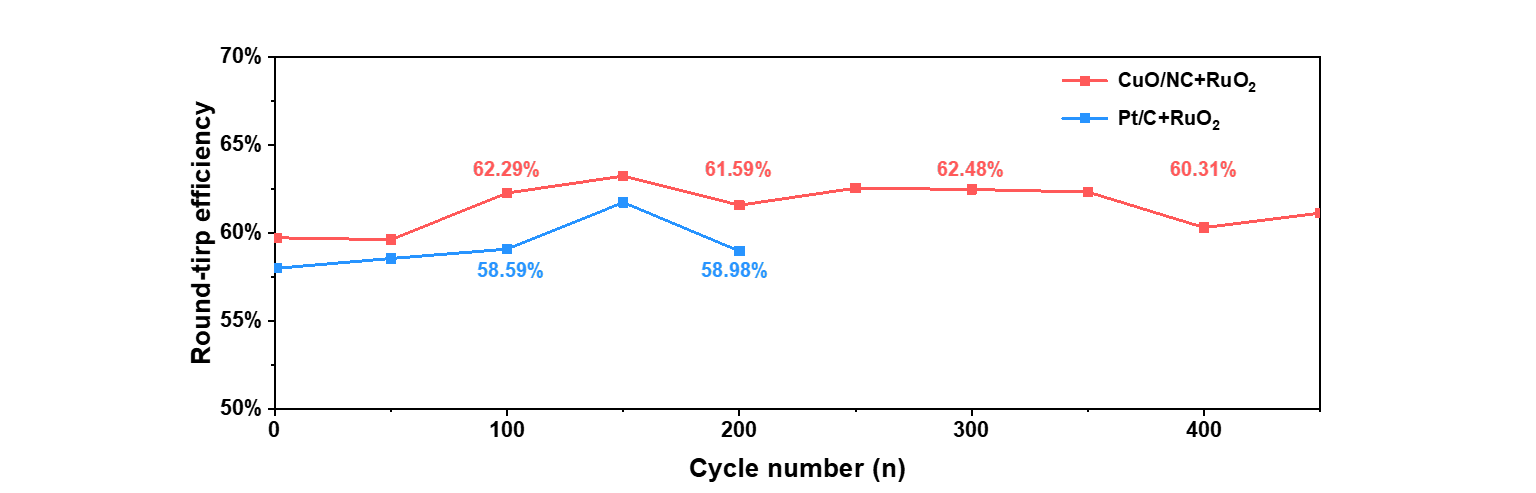


**Figure S26.** The Round-trip efficiency of CuO/NC+RuO_2_ and Pt/C+RuO_2_ based ZABs.


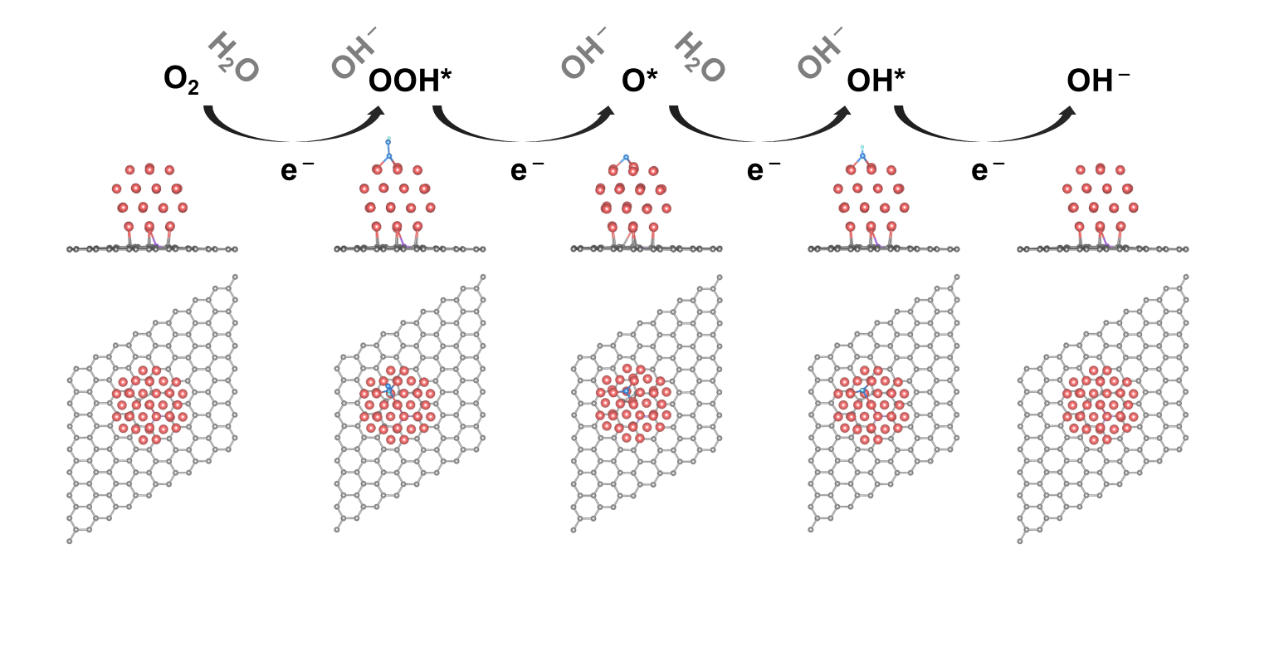


**Figure S27.** The ORR reaction pathway and intermediates adsorption structure of *OOH, *O, and *OH on the pyridinic nitrogen-doped carbon-supported Cu NPs.


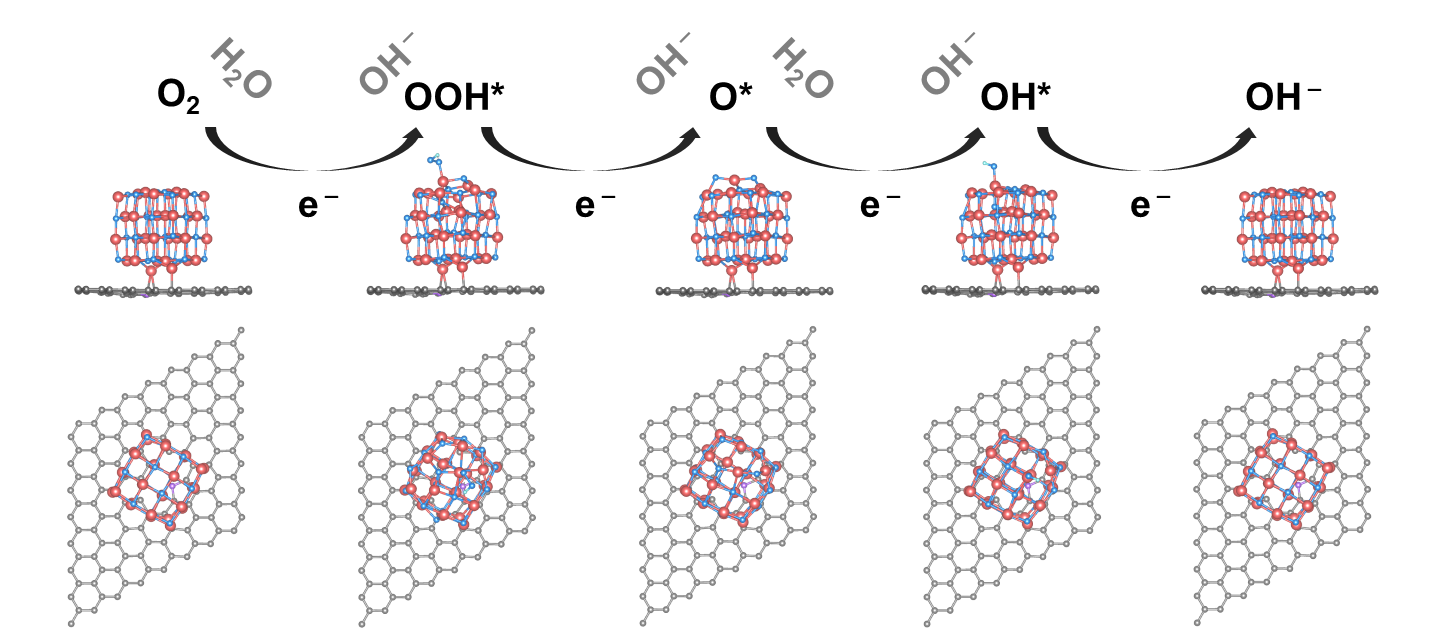


**Figure S28.** The ORR reaction pathway and adsorption intermediates structure of *OOH, *O, and *OH on the pyrrole nitrogen-doped carbon-supported CuO.


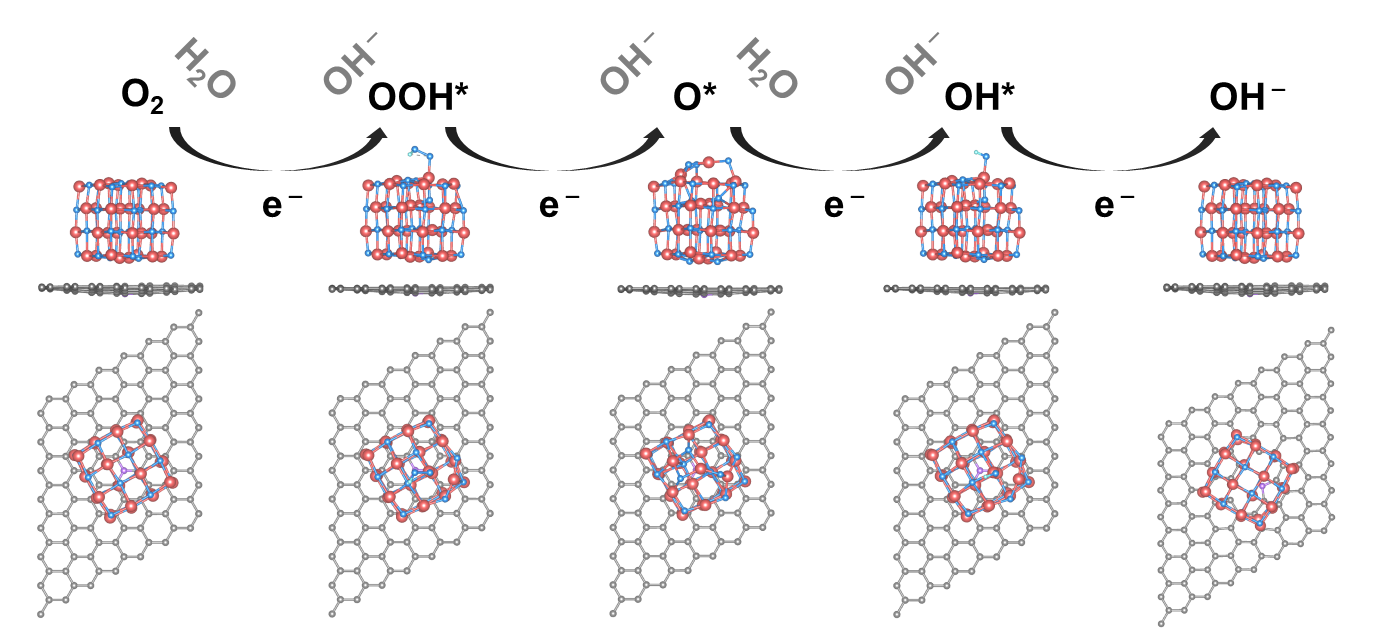


**Figure S29.** The ORR reaction pathway and adsorption intermediates structure of *OOH, *O, and *OH on the graphene nitrogen-doped carbon-supported CuO.

**Figure S30.** The calculated Gibbs free energy diagrams of ORR on pyridinic, pyrrole, graphene nitrogen-doped carbon-supported CuO, respectively.


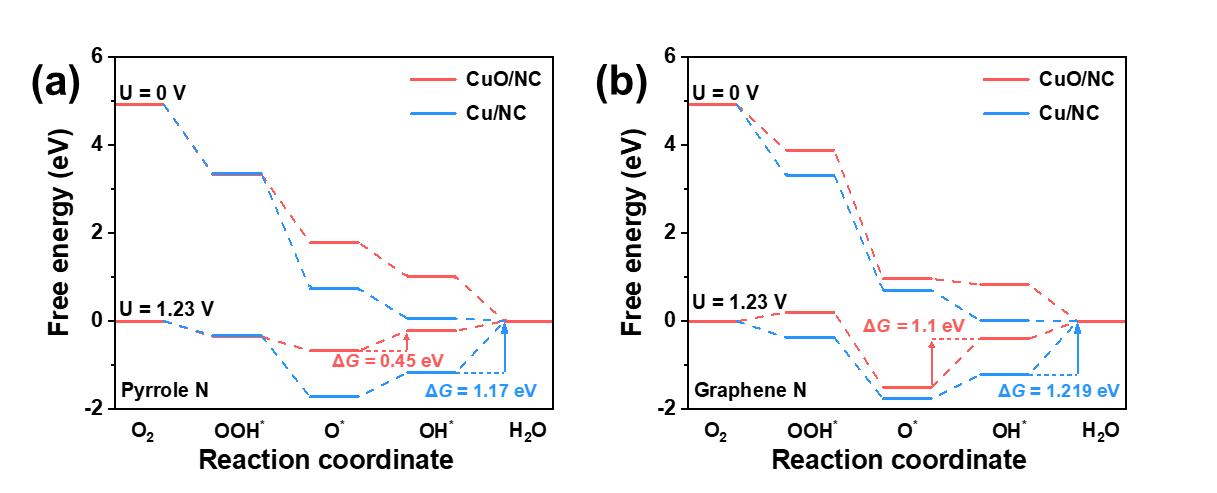


**Figure S31.** The calculated Gibbs free energy diagrams of ORR on (a) pyrrole nitrogen-based CuO/NC and Cu/NC, (b) graphene nitrogen-based CuO/NC and Cu/NC.


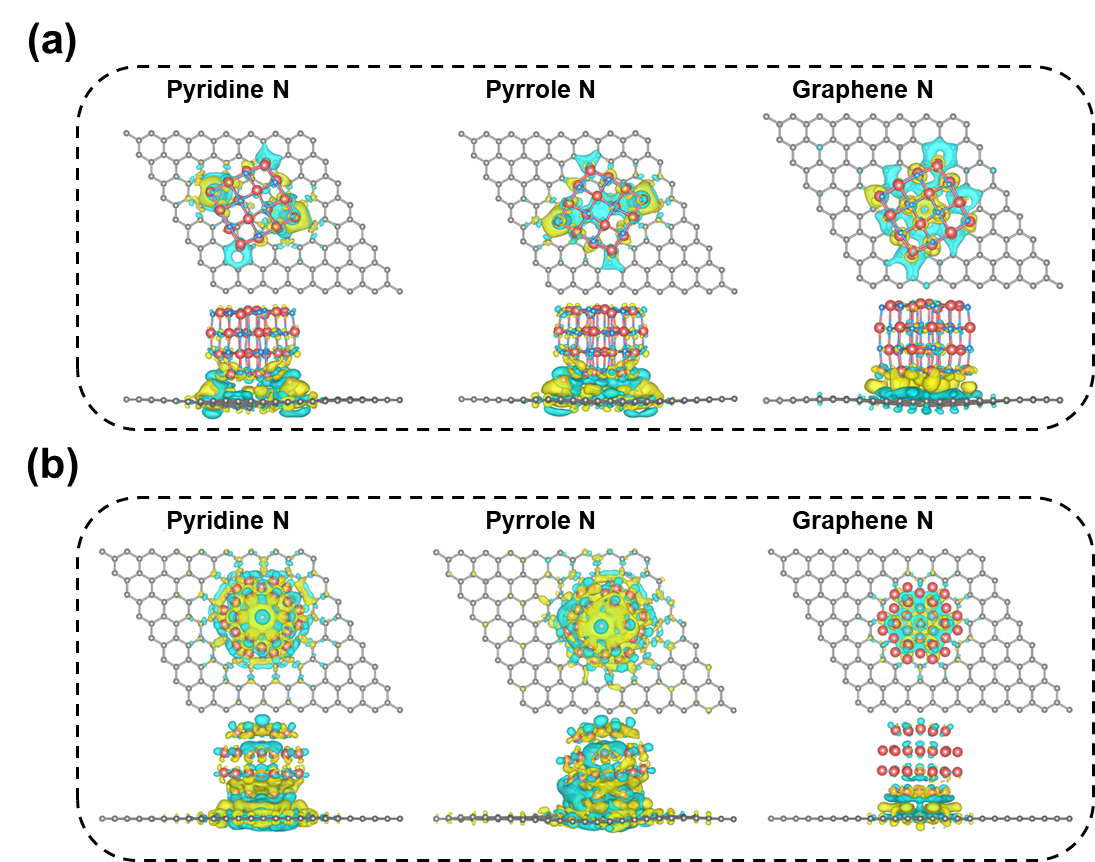


**Figure S32.** The difference charge density plots of (a) CuO/NC and (b) Cu/NC, the deletion and accumulation of electrons are represented by the blue and yellow contours, respectively. (The iso-surface corresponds to 0.0005 e Å^−3^)

**Supporting Tables**

**Table S1.** BET specific surface areas and pore volume of the various catalysts.

| Properties | CuO/NC | Cu/NC | NC-900 |
| --- | --- | --- | --- |
| Specific surface area (m^2^ g^-1^) | 383 | 414 | 704 |
| Pore volume (cm^3^ g^-1^) | 0.89 | 0.86 | 1.17 |
| Average pore size (nm) | 9.3 | 8.3 | 6.7 |

**Table S2.** The different chemical states of N content in CuO/NC and Cu/NC.

| Samples | pyridinic N | Cu-N*_x_* | pyrrolic N | graphitic N | oxidized N |
| --- | --- | --- | --- | --- | --- |
| CuO/NC | 47.66% | 13.98% | 25.43% | 5.30% | 6.63% |
| Cu/NC | 40.75% | 19.75% | 22.44% | 8.08% | 8.98% |

**Table S3.** EXAFS fitting parameters at the Cu K-edge for various samples (*Ѕ*_0_^2^=0.84).

|  | shell | CN | R(Å) | σ^2^ | ΔE_0_ | R factor |
| --- | --- | --- | --- | --- | --- | --- |
| Cu foil | Cu-Cu | 12* | 2.54±0.01 | 0.0087 | 4.4±0.6 | 0.0035 |
| CuO/NC | Cu-O | 3.9±0.1 | 1.94±0.01 | 0.0059 | 5.5±0.9 | 0.0088 |
|  | Cu-Cu | 1.2±0.3 | 2.91±0.02 | 0.0099 |  |  |

*^a^N*: coordination numbers; *^b^R*: bond distance; *^c^σ*^2^: Debye-Waller factors; *^d^* Δ*E*_0_: the inner potential correction. *R* factor: goodness of fit.

**Table S4.** Density of States on the Fermi level of graphitic N, pyrrole N, pyridine N-based CuO/NC and Cu/NC.

| Materials | DOS | | |
| --- | --- | --- | --- |
|  | graphitic N | pyrrolic N | pyridinic N |
| CuO/NC | 9.27 | 20.76 | 21.26 |
| Cu/NC | 11.69 | 5.03 | 5.78 |

**Table S5.** Calculated the Bader charge of the *OH adsorbed on the Cu atom of CuO/NC and Cu/NC with graphitic N, pyrrolic N and pyridinic N doped.

| Materials | Bader charge (e) | | |
| --- | --- | --- | --- |
|  | graphitic N | pyrrolic N | pyridinic N |
| CuO/NC | 0.45 | 0.46 | 0.46 |
| Cu/NC | 0.6 | 0.61 | 0.61 |

**Table S6.** Comparison of ORR activity of CuO/NC and Cu/NC with recently reported Cu-based catalysts.

| Materials | *E*_1/2_  (V vs.RHE) | Tafel slope  (mV dec^-1^) | Ref. |
| --- | --- | --- | --- |
| **CuO/NC** | **0.873** | **53.08** | **This work** |
| **Cu/NC** | **0.857** | **57.80** |
| Cu-ZrO_3-x_@N-BPCNFs | 0.856 | 85 | *Small* **2023**, *19*, 2206823 |
| SA-CoCu@Cu/CoNP | 0.88 | 59.9 | *Adv. Energy Mater.* **2021**, *11*, 2100303 |
| (Zn, Cu)-NC | 0.88 | 87.1 | *Adv. Funct. Mater.* **2022**, *32*, 2203471 |
| Cu SAC/P-700 | 0.87 | 72.4 | *Appl. Catal. B-Environ.*  **2023**, *338*, 123043 |
| CuSA/g-C_3_N_4_-1000 | 0.85 | 57 | *J. Am. Chem. Soc.* **2023**, *145*, 27054 |
| 2.0 wt % Cu-N-C SAC | 0.83 | 37 | *J. Am. Chem. Soc.* **2021**, *143*, 14530 |
| Cu/Zn-NC | 0.83 | 54.8 | *Angew. Chem. Int. Ed.* **2021**, *60*, 14005 |
| Cu-N©HCS | 0.812 | 32.4 | *Small* **2023**,*19*, 2301327 |
| Cu NDs/Fe_2_O_3_-NPCs | 0.85 | 53.70 | *Chem. Eng. J.* **2022**, *442*, 136128 |
| Cu-0.25 V/SNGF-0.4 | 0.866 | 75.3 | *Appl. Catal. B-Environ.*  **2021**, *289*, 120028 |
| Cu-N-C/GC | 0.84 | 64.9 | *Angew. Chem. Int. Ed.* **2022**, *61*, e202211098 |
| CoS_2_/Cu_2_S-NF | 0.80 | 88.06 | *Appl. Catal. B-Environ.*  **2022**, *303*, 120849 |

**Table S7.** Comparison of Zn-air battery performances of CuO/NC with recently other benchmarking counterpart’s electrode materials reported in literatures.

| Materials | OCV  (V vs. Zn) | Specific capacity  (mAh g^–1^) | Maximum power density  (mW cm^–2^) | Ref. |
| --- | --- | --- | --- | --- |
| **CuO/NC** | **1.51** | **787.14 (10 mA cm^-2^)** | **255.4** | **This work** |
| Pt_1_–Fe/Fe_2_O_3_ | 1.53 | 778 (10 mA cm^-2^) | 182 | *Nat. Energy* **2021**, *6*, 614 |
| Pt=N_2_=FeABA | 1.50 | 787.8 (10 mA cm^-2^) | 198.4 | *Nat. Commun.* **2022**, *13*, 6414 |
| FeMn_ac_/Mn-N_4_C | 1.46 | 720.2 (10 mA cm^-2^) | 207 | *Angew. Chem. Int. Ed.* **2023**, *62*, e202214988 |
| SA-Fe-SNC@900 | 1.464 | 772.9 (10 mA cm^-2^) | 218.6 | *Adv. Mater.* **2023**, *35*, 2209948 |
| Pd−Gd_2_O_3_/C | 1.46 | 724 (5 mA cm^-2^) | 111.1 | *Angew. Chem. Int. Ed.* **2023**, *62*, e202314565 |
| Co_2_/Fe–N@CHC | 1.499 | 786.1 (10 mA cm^-2^) | 232.4 | *Adv. Mater.* **2021**, *33*, 2104718 |
| BP-CN-c | 1.47 | 793.9 (5 mA cm^-2^) | 168.3 | *Adv. Mater.* **2021**, *33*, 2008752 |
| NiCo_1.8_Fe_0.2_O_4_@NCF | 1.51 | 802 (10 mA cm^-2^) | 180 | *Angew. Chem.Int. Ed.* **2024**, *63*, e202319983 |
| Ce SAs/PSNC | 1.49 | 783 (10 mA cm^-2^) | 212 | *Adv. Mater.* **2023**, *35*, 2302485 |
| CR-Co/ClNC | 1.50 | 745 (10 mA cm^-2^) | 176.6 | *Nat. Commun.* **2024**, *15*, 1675 |

References

[1] F. Gándara, F. J. Uribe-Romo, D. K. Britt, H. Furukawa, L. Lei, R. Cheng, X. Duan, M. O'Keeffe, O. M. Yaghi, *Chem.-Eur. J.* **2012**, *18*, 10595.

[2] G. Kresse, J. Furthmüller, *Comput. Mater. Sci.* **1996**, *6*, 15.

[3] G. Kresse, J. Furthmüller, *Phys. Rev. B* **1996**, *54*, 11169.

[4] J. P. Perdew, K. Burke, M. Ernzerhof, *Phys. Rev. Lett.* **1996**, *77*, 3865.

[5] S. Grimme, J. Antony, S. Ehrlich, H. Krieg, *J. Chem. Phys.* **2010**, *132*, 154104.

[6] W. Xue, Q. Zhou, X. Cui, J. Zhang, S. Zuo, F. Mo, J. Jiang, X. Zhu, Z. Lin, *Angew. Chem. Int. Ed.* **2023**, *62*, e202307504.

[7] J. Lan, Z. Wei, Y.-R. Lu, D. Chen, S. Zhao, T.-S. Chan, Y. Tan, *Nat. Commun.* **2023**, *14*, 2870.
